# Catabolism of Exogenous Lactate Reveals It as a Legitimate Metabolic Substrate in Breast Cancer

**DOI:** 10.1371/journal.pone.0075154

**Published:** 2013-09-12

**Authors:** Kelly M. Kennedy, Peter M. Scarbrough, Anthony Ribeiro, Rachel Richardson, Hong Yuan, Pierre Sonveaux, Chelsea D. Landon, Jen-Tsan Chi, Salvatore Pizzo, Thies Schroeder, Mark W. Dewhirst

**Affiliations:** 1 Department of Pathology, Duke University Medical Center, Durham, North Carolina, United States of America; 2 Department of Radiation Oncology, Duke University Medical Center, Durham, North Carolina, United States of America; 3 Duke Cancer Institute, Duke University Medical Center, Durham, North Carolina, United States of America; 4 Duke University Shared Resources NMR Facility, Duke University, Durham, North Carolina, United States of America; 5 Institute of Genome Sciences and Policy, Duke University Medical Center, Durham, North Carolina, United States of America; 6 Department of Molecular Genetics and Microbiology, Duke University Medical Center, Durham, North Carolina, United States of America; 7 Department of Radiology, University of North Carolina, Chapel Hill, North Carolina, United States of America; 8 Pole of Pharmacology, Institut de Recherches Expérimentales et Cliniques (IREC), Université catholique de Louvain (UCL), Brussels, Belgium; Instituto Nacional de Cardiologia, Mexico

## Abstract

Lactate accumulation in tumors has been associated with metastases and poor overall survival in cancer patients. Lactate promotes angiogenesis and metastasis, providing rationale for understanding how it is processed by cells. The concentration of lactate in tumors is a balance between the amount produced, amount carried away by vasculature and if/how it is catabolized by aerobic tumor or stromal cells. We examined lactate metabolism in human normal and breast tumor cell lines and rat breast cancer: 1. at relevant concentrations, 2. under aerobic vs. hypoxic conditions, 3. under conditions of normo vs. hypoglucosis. We also compared the avidity of tumors for lactate vs. glucose and identified key lactate catabolites to reveal how breast cancer cells process it. Lactate was non-toxic at clinically relevant concentrations. It was taken up and catabolized to alanine and glutamate by all cell lines. Kinetic uptake rates of lactate *in vivo* surpassed that of glucose in R3230Ac mammary carcinomas. The uptake appeared specific to aerobic tumor regions, consistent with the proposed “metabolic symbiont” model; here lactate produced by hypoxic cells is used by aerobic cells. We investigated whether treatment with alpha-cyano-4-hydroxycinnamate (CHC), a MCT1 inhibitor, would kill cells in the presence of high lactate. Both 0.1 mM and 5 mM CHC prevented lactate uptake in R3230Ac cells at lactate concentrations at ≤20 mM but not at 40 mM. 0.1 mM CHC was well-tolerated by R3230Ac and MCF7 cells, but 5 mM CHC killed both cell lines ± lactate, indicating off-target effects. This study showed that breast cancer cells tolerate and use lactate at clinically relevant concentrations *in vitro* (± glucose) and *in vivo.* We provided additional support for the metabolic symbiont model and discovered that breast cells prevailingly take up and catabolize lactate, providing rationale for future studies on manipulation of lactate catabolism pathways for therapy.

## Introduction

Normal physiologic range of lactate concentration in the blood is ∼ 0.5–2 mM [1]; in contrast, pathophysiologic lactate concentrations in tumors range from normal lactate levels to concentrations as high as 40 mM [2]. In the 1920s Otto Warburg was the first to discover that tumors accumulate excess lactate [3]–[5]. In the last hundred years, the importance of this metabolic switch in tumor tissue has become increasingly evident, and, recently, elevated lactate levels in tumors has been coined as a hallmark of cancer by Hanahan and Weinberg [6].

Lactate accumulation within tumor tissue is mainly due to the increased glycolytic rate of cancer cells. This increase in glycolysis is in response to a number of factors: hypoxia (Pasteur Effect), proliferative demand, increased oxidative stress and altered genetic programming [7]–[9]. Increases in lactic acid in tumors combined with lack of buffering capacity contribute to localized areas of low pH in tumors [7], [8]. It has been observed that lactate accumulation is correlated with hypoxia in some tumor types [10] (Pasteur Effect), and, clinically, hypoxia is correlated with poor patient prognosis and survival [11], [12]. However, high lactate is not a surrogate marker of hypoxia. Studies of genomic regulation by hypoxia vs. lactate vs. acidosis in cancer cells showed that lactate regulated a different set of genes than hypoxia [13]. The consequences of downstream lactate signaling in normal mammary epithelial cells exposed to high lactate showed repression of glycolytic genes. In several large breast cancer clinical series where gene expression data were available, the “lactic acidosis” genomic signature with repressed glycolysis was associated with significantly increased patient survival rates [13]. This indicates that the response of the tumor to high lactate is important to patient outcome and that lactate utilization and catabolism by the tumor warrants investigation in order to understand how cancer cells cope with high lactate concentrations.

Monocarboxylate transporters (MCTs) facilitate movement of lactate in and out of the cell. There are 14 different subtypes, four of which are relatively well-characterized: MCT1, MCT2, MCT3 and MCT4 [14], [15]. Of these, MCT1 is the most ubiquitously expressed subtype. MCT1 inhibition has been receiving attention as a potential anti-cancer treatment option [16], [17]. We previously reported that lactate can serve as an energy source for aerobic cells and proposed a “metabolic symbiont” model within the tumor microenvironment. In this model, lactate produced by hypoxic cells can provide an additional substrate for aerobic cells. With the aerobic cells utilizing the lactate for energy, they will utilize less glucose, thereby allowing some glucose to reach the hypoxic cells [17]. We found that SiHa (cervical cancer) cells, which expressed higher levels of MCT1 but lower levels of MCT4, consumed significantly more lactate and less glucose than WiDr (colorectal cancer) cells. Conversely, WiDr cells, which expressed higher levels of MCT4 and lower levels of MCT1, consumed less lactate and more glucose than SiHa cells [17]. Recently, MCT subtype and LDH isoform expression has been characterized in HMEC, MCF7 and MDA-MB-231 cells [18]. HMEC display the greatest amount of MCT1 expression on the cell membrane and express both LDHA and LDHB. MCF7 cells display MCT1 expression on the cell membrane in lower levels than HMEC and express both LDHA and LDHB. MCF7 cells exhibit higher LDHB expression than MDA-MB-231 cells. MDA-MB-231 cells do not express MCT1. They express both LDHA and LDHB, with higher LDHA than MCF7 cells. This suggests that there is a connection between MCT subtype expression and a lactate-consuming ability in cancer cells. Given these differences of expression of MCT subtypes [18] and our previous findings of lactate consumption in connection with MCT subtype expression [17], we hypothesized that lactate uptake and catabolism would be different between the breast cells.

Lactate transport can be manipulated by MCT-inhibitors [17]. The small molecule MCT-inhibitor α-cyano-4-hydroxycinnamate (CHC) is >10 fold more selective for inhibition of MCT1 than for inhibition of MCT4 [19]. It was proposed that inhibition of MCT1 by CHC or knockdown of MCT1 using siRNA would prevent lactate uptake in the aerobic cells, forcing them to utilize glucose, thereby starving the more treatment-resistant hypoxic cells [17]. In cell-based assays it was shown that CHC decreases lactate-fueled respiration and ATP production in both SiHa and WiDr cells [17]. It was also shown that treatment with CHC significantly decreased tumor growth similar to siMCT1 in xenograft models [17]. These results warrant further investigation of MCT1 inhibition as an anti-tumor treatment option. It has already been reported that MCT1 inhibition can lead to cancer cell death via a lethal decline in pH_i_ with blockade of endogenous lactic acid exportation [20]. We hypothesized that pharmacological inhibition of exogenous lactate metabolism with CHC could elicit cell death by preventing exogenous lactate entry and utilization in glucose-deprived conditions.

Our cell-based studies focused on four cell lines: Human mammary epithelial cells (HMEC), MCF7 (human mammary adenocarcinoma), MDA-MB-231 (human mammary adenocarcinoma), and R3230Ac (rat mammary carcinoma) cells. HMEC cells were included to compare a normal cell response to exogenous lactate with cancer cell responses to lactate. Catabolism studies were also conducted on HUVEC cells as a second normal tissue line. The R3230Ac rat mammary carcinoma was used as our *in vivo* model, because we have studied glucose uptake and its conversion to lactate previously in this model previously [21], [22]. Both MCF7 and MDA-MB-231 cells were included as breast cancer models for two primary reasons. First, we wanted to represent a luminal (MCF7) and a basal-like (MDA-MB-231) breast cancer subtype [23], as these subtypes are known to be considerably different clinically and pathologically [23]-[25]. Second, it has previously been reported that MCT1 is silenced in MDA-MB-231 cells [18], [26]. By including both MDA-MB-231 and MCF7 cells in our experiments, we could compare lactate uptake and metabolism in breast cancer cells lacking and expressing MCT1. Though our studies did not focus on p53 signaling and lactate metabolism, it is important to mention that the p53 status in each of these cell lines differ considerably: R3230Ac and MCF7 cells are p53 WT [27]–[29] while MDA-MB-231 cells are p53 null [30], [31]. This may be an important avenue for future investigation because p53 influences many metabolic pathways including glycolysis, oxidative phosphorylation and mTOR signaling [32], [33].

In this study, we expanded on our prior work on lactate metabolism, focusing on breast cancer. The main goals were: 1) to establish the tolerance of breast cancer cells to a range of lactate concentrations typical of that seen in human breast cancer, 2) to investigate lactate catabolism *in vitro* and *in vivo* and 3) to examine whether treatment with CHC elicits cell death in a lactate-dependent manner. We used two doses of CHC sufficient to inhibit MCT1; one concentration chosen (5 mM) was based on previous studies, and the other concentration chosen was based on reported *K*_i_ values for the compound [14], [17]. To investigate lactate metabolism *in vivo*, we employed the R3230Ac tumor model, which has been shown to exhibit regions of high lactate in the absence of measureable glucose [10].

## Results

### Lactate accumulation occurs in locally advanced breast cancer (LABC) with a median concentration range of 0.6 – 8.0 µmol/g, and lactate accumulation shows high intra-tumoral variation

Lactate concentrations have been measured in human head and neck [34], cervical [2] and colorectal [35] cancers by bioluminescent technology. Lactate levels in breast cancer have not previously been measured. We sought to define the range of lactate concentrations found in LABC, to guide our cell-based assays. Twenty-three frozen breast-core biopsies from 21 patients with locally advanced breast cancer (LABC) (two biopsies were from the same patient, different sites of the tumor) were made available to us from an Institutional Review Board (IRB)-approved phase I/II clinical trial conducted at Duke University (Table 1).

**Table 1:** Patient clinical parameters and lactate levels in LABC biopsies.

Group	Sample #	% Invasive cancer	Outcome	TMN	Distant Mets.	Relapse	Age	[La] 5PMin	[La] 25P	[La] 50PMed.	[La] 75P	[La] 95PMax
**Lo [L]**	bb1	0	alive	2	n	n	39	0.0	0.0	0.8	1.5	2.6
	LABC1	70	alive	2	n	n	62	0.0	0.0	0.6	1.3	2.5
	bb3	0	alive	3	n	n	54	0.6	1.7	2.5	3.9	4.8
	LABC2	30	alive	3	n	n	62	0.3	1.5	2.6	3.5	5.2
	LABC3	70	alive	3	n	n	55	0.6	1.8	2.9	3.8	5.4
	LABC4	95	alive	**4**	**y**	n	32	0.4	1.8	3.3	3.7	5.4
	LABC6	80	**dead**	**4**	**y**	n	63	1.1	2.5	3.8	4.9	7.0
	LABC7	75	**dead**	**4**	**y**	**y**	58	1.2	2.7	4.0	5.2	7.2
	LABC8	75	alive	3	n	n	55	1.4	2.9	4.2	5.3	7.5
	LABC9	70	alive	3	n	n	43	1.4	3.0	4.2	5.5	7.7
	LABC710(d)	65	alive (d)	2 (d)	n (d)	n (d)	62 (d)	1.7	3.1	4.3	5.3	7.1
	bb4	0	alive	2	n	n	35	0.7	2.5	4.4	5.5	7.8
**Hi [L]**	LABC11	70	**dead**	**4**	**y**	**y**	75	1.6	3.1	4.5	5.7	7.8
	LABC12	75	**dead**	**4**	**y**	?	66	0.9	2.6	4.5	5.1	7.2
	LABC13	80	alive	2	n	n	44	1.5	3.2	4.6	5.7	7.8
	LABC14	60	alive	?	?	?	?	1.5	3.3	5.0	6.3	8.9
	LABC15	95	alive	3	n	n	35	1.0	2.9	5.1	5.7	8.6
	LABC16	80	**dead**	3	n	y	32	1.6	3.1	5.2	6.0	8.2
	LABC17	80	alive	3	n	y	49	2.0	3.7	5.2	6.6	8.9
	LABC18	80	**dead**	2	n	n	27	1.8	3.8	5.6	7.0	9.4
	LABC19	75	alive	**4**	**y**	n	52	2.9	4.9	6.6	8.1	10.9
	LABC20	80	alive	3	n	n	43	3.3	5.3	7.2	8.8	12.3
	LABC21(d)	90	alive (d)	3 (d)	n (d)	n (d)	35 (d)	4.0	6.0	8.0	9.4	12.2

Specimens are named based on presence of benign breast tissue (bb) or presence of invasive cancer (LABC). Duplicate specimens are indicated (d). Measured lactate concentrations (μmol/g of tissue) are displayed in the last five columns. These values correspond to the lactate concentration range ([L]) seen in microregions in each specimen: fifth percentile/minimum (5P), twenty-fifth percentile (25P), fiftieth percentile/median (50P), seventy-fifth percentile (75P) and ninety-fifth percentile/maximum (95P). Samples are arranged in ascending order of median lactate concentrations. High and low lactate groups were determined based on median lactate concentrations >4.4 µmol/g. LABC5 and bb2 specimens are missing due to unavailable clinical information on these patients.

**Figure 1:**
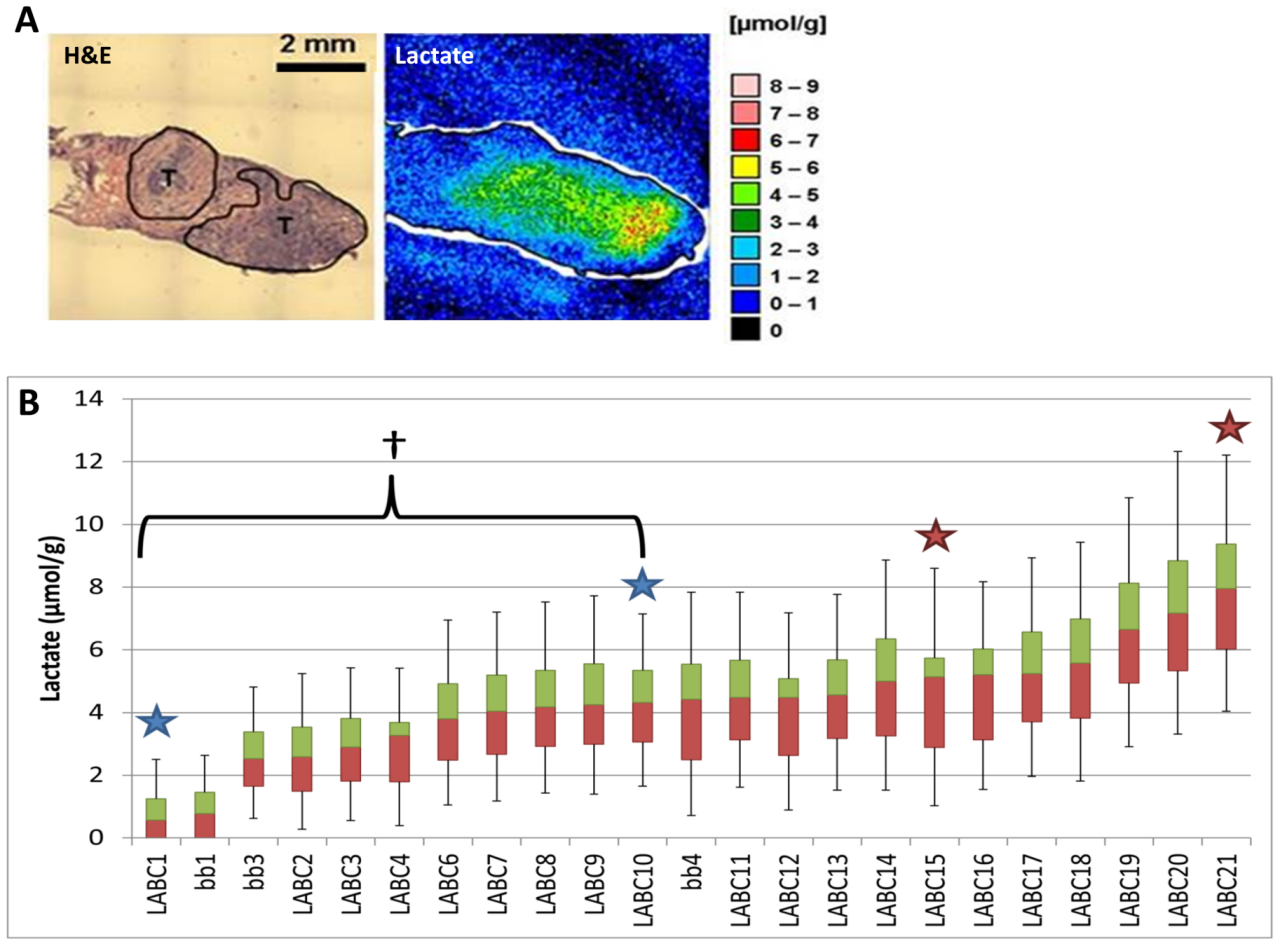
Human tumor samples measured for lactate with bioluminescence *ex vivo* show lactate concentrations vary considerably between samples. Bioluminescence color map for lactate concentrations measured in a LABC patient biopsy (A). Tumor regions are outlined and marked (T). Waterfall Box & Whisker plot for lactate concentrations (μmol/g) measured in benign breast tissue (bb) and LABC patient biopsies (B). Stars indicate separate biopsies from the same patient. LABC1 and LABC10 samples show a significant difference in lactate concentrations (†p<0.05, Student’s T-test). First quartile values represented in red; third quartile values are represented in green. Median values represented at the interface of red and green boxes. Error bars represent the 5^th^ and 95^th^ percentile lactate concentrations. All biopsies n = 3–4.

Lactate concentrations were measured with bioluminescence imaging [10], [36], [37] (Fig 1A). The median lactate concentration for the entire sample set was 4.4 µmol/g. Samples could be divided into low v. high lactate groups based on this median (Table 1). The range of median lactate values for LABC biopsies was 0.6 to 8.0 µmol/g, and the median range for benign breast tissue (“bb”) was 0.8 – 1.9 µmol/g (Fig 1B). The quartile range and 95% confidence intervals demonstrated a wide range of lactate values; the 95^th^ percentile showed lactate levels greater than12 µmol/g (Fig 1B). These results illustrate the heterogeneity in lactate accumulation within individual tumors and between tumors. However, these concentrations were considerably lower than seen in other tumor types. For example, in head and neck cancer, median values were in the range of 7 µmol/g with microregional variation extending as high as 40 µmol/g [2], [34]. Two of three biopsies that did not have any invasive cancer (“bb”) showed lower lactate accumulation than the majority of biopsies with invasive cancer (Fig 1B).

The two specimens that represented second biopsy site from the same patient (stars, Fig 1B) fell into the same high or low lactate group as the first biopsy site (Table 1). Specimens LABC1 and LABC10 are biopsies from the same patient and are in the low lactate group, while specimens LABC15 and LABC21 (Fig 1B) are from another patient and are in the high lactate group (second site indicated by “d” in Table 1). The median lactate concentrations measured in LABC1 and LABC10 (0.6 and 4.3 µmol/g) were significantly different (Student’s T-test, p < 0.05), while the median lactate concentrations measured in LABC15 and LABC21 (5.1 and 8.0 µmol/g, respectively) were not (Fig 1B, Table 1). From these dual biopsy sites from one tumor, the intra-tumor metabolic heterogeneity is illustrated, indicating that lactate levels can vary significantly among different areas of the same tumor.

### Lactate uptake and metabolism occurs *in vitro* in R3230Ac cells

**Figure 2:**
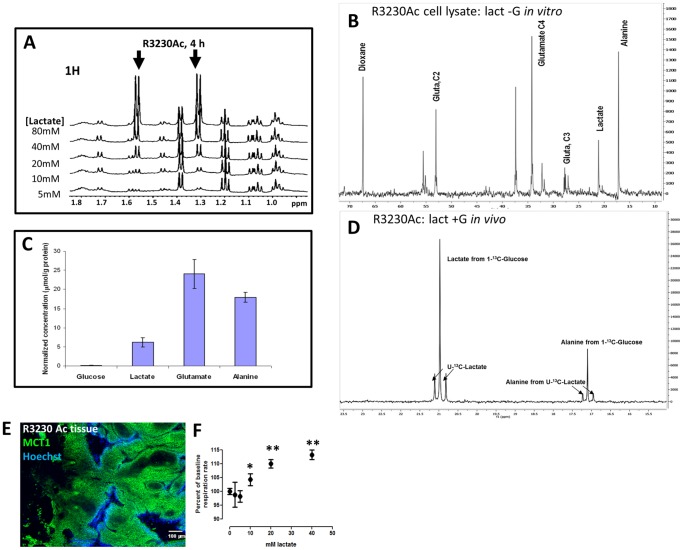
R3230Ac cells take up and metabolize lactate to alanine and glutamate *in vitro* and *in vivo.* ^1^H NMR of R3230Ac cell lysates exposed to various concentrations of lactate for 4 h (glucose absent) showed a concentration-dependent lactate uptake (arrows, ∼1.3 and ∼1.6 ppm, **A**). ^13^C NMR plot for R3230Ac cell lysate after 12 h treatment with 5 mM 3-^13^C-lactate (no glucose) showing generation of ^13^C-labeled glutamate and alanine species (**B**). Dioxane was included as an internal standard which allowed quantification of labeled metabolites (**C**). ^13^C NMR plot from R3230Ac tumor after infusion with equimolar concentrations of universally labeled U-^13^C-lactate and 1-^13^C-glucose showing uptake of U-^13^C-lactate (∼20.75 and ∼21.25 ppm) and generation of U-^13^C-alanine species (∼16.9 and 17.2 ppm) in the presence of labeled glucose and during production of glycolytically-derived lactate (∼21 ppm) and alanine (∼17.1 ppm) (**D**). Tissue staining of R3230Ac tumor shows positive expression of MCT1 (green); areas of perfusion are indicated by Hoechst 33342 (blue) (**E**). Glucose-deprived R3230Ac cells show significantly increased oxygen consumption (n = 3, Student’s T-test, p < 0.05) with increasing concentrations of exogenous lactate compared to the untreated control (**F**).

Before studying lactate metabolism in cells it was important to assess cell viability after lactate treatment to ensure that the metabolic results would not be skewed by dying cells. The range of lactate concentrations tested *in vitro* was defined by our experimental findings in LABC (Fig 1) and the literature on lactate accumulation in tumors [2], [22], [34], [35], [38]–[40]. Concentrations of 5 and 10 mM lactate reflect the concentrations found in the LABC biopsies and the median lactate concentration found in other solid tumors, such as head and neck and cervical cancer [2], [34], [38]. It was previously published that microregions of some tumors can reach up to 40 mM lactate [2], which we defined as our upper limit. 20 mM lactate was included in some experiments as an intermediary concentration between the low and high limits; however, this concentration was still higher than what was found in the clinical LABC specimens. The acute effects of lactate toxicity were examined using Annexin V and 7-aminoactinomycin D (7-AAD) to assess apoptosis and membrane integrity, respectively, in cells exposed to lactate (0, 10, 20 and 40 mM) for 24 h (Fig S1 and S2). These studies were conducted in the presence (Fig S1) and absence of glucose (Fig S2) because it has been reported that lactate accumulation can occur in the presence or absence of glucose *in vivo* [10]. Additionally, we chose to use glucose-free media for a majority of our NMR studies in order to acquire higher signals for lactate and lactate-generated metabolites. When exogenous sodium lactate was supplemented in cell culture media containing glucose, none of the cells showed any decrease in cell viability or increase in cell death responses (Fig S1). Likewise, all cell lines treated with 0 –20 mM in glucose-deprived conditions survived (Fig S2). Only at 40 mM lactate (- glucose) did MCF7 and MDA-MB-231 cells show significant cell death responses (FigS2F&G). Glucose deprivation usually elicits cell stress responses, such as activation of JNK1 and increased oxidative stress [41]–[45]. This may suggest that very high lactate concentrations can augment the cellular stress elicited by glucose deprivation, but 40 mM lactate has been reported very infrequently in solid tumors and concentrations this high were not found in any of the breast tumor biopsies evaluated in this study [2], [10], [34], [38], [39], [46], [47]. Thus, we conclude that cell viability is not appreciably affected over the range of physiologically relevant lactate concentrations observed in breast cancer (Fig 1) with or without glucose.

We hypothesized that normal breast cells and breast cancer cells could utilize exogenous lactate for metabolic purposes. We used NMR with ^13^C-labeled lactate to track uptake; we first focused on the lactate metabolism in R3230Ac cells. *In vitro*, R3230Ac cells took up lactate in a concentration-dependent manner after 4 h of treatment (Fig 2A; lactate peaks indicated by arrows). In glucose-deprived conditions, R3230Ac cells were treated with 5 mM 3-^13^C-lactate for 12 h. The ^13^C spectra of the cell lysate show peaks corresponding to ^13^C-lactate, ^13^C-alanine and ^13^C-glutamate (Fig 2B). When these metabolites were normalized to protein levels, glutamate was the most abundant (Figure 2C). To determine if lactate would be metabolized in the presence of glucose, 5 mM concentrations of U-^13^C-lactate and 1-^13^C-glucuose were infused in tumor-bearing rats. The utilization of universally labeled lactate allows one to distinguish the uptake and metabolism of lactate from the blood as opposed to lactate and other metabolites produced from glucose. In Figure 2D, the first three peaks are for lactate, and the next three peaks are for alanine. Due to the different position of ^13^C, the lactate metabolized from 1-^13^C-glucose is separated from universally labeled lactate; a similar pattern emerges with alanine metabolized from lactate or glucose. The ratio of universally labeled lactate taken up by the tumor versus lactate produced from glucose was 0.36. Additionally, alanine is produced from both glucose and lactate. The results indicate that the tumor takes up lactate even in the presence of glucose. This is the first time a breast tumor has been shown to simultaneously take up lactate and glucose and metabolize both substrates *in vivo*.

Lactate transport is dependent upon expression of proton-coupled, lactate-specific transporters of the monocarboxylate transporter (MCT) family. MCTs are passive, bidirectional transporters with different K_m_ constants for lactate(MCT1  =  ∼3–5 mM, MCT2  =  ∼0.7 mM and MCT4  =  ∼28 mM) [14]. MCT1 has ubiquitous tissue distribution but is found to be upregulated in cancer [48]–[51]. MCT4 has a more specific tissue distribution; it is associated primarily with glycolytic cells/tissues and is regulated by the hypoxia inducible transcription factor, HIF-1 [52]–[54]. Previously, we have shown that cancer cell lines with high MCT1/low MCT4 expression consume more lactate than cancer cell lines with low MCT1/high MCT4 expression [17].

Although it is well-documented that most normal cells and cancer cells typically express MCT1 [14], [48]–[51], MCT1 expression in R3230Ac cells has not been tested previously. R3230Ac tumor shows abundant membrane expression of MCT1 (Fig 2E), while MCT4 was undetectable (Fig S3B), consistent with a lactate-consuming phenotype. It has previously been reported that R3230Ac cells utilize oxidative phosphorylation as well as glycolysis [55]. Glutamate was found to be the predominant lactate-derived metabolite produced in R3230Ac cells *in vitro* (Fig 2B). Glutamate is a TCA cycle by-product, formed from α-ketoglutarate. This indicates that one pathway of lactate metabolism is respiration. In addition to glutamate formation indicating cellular respiration of lactate, R3230Ac cells were treated with increasing concentrations of lactate (-glucose) *in vitro,* and oxygen consumption rate was measured. All concentrations of lactate ≥ 10 mM tested showed a significant increase in oxygen consumption rate in the cells (Fig 2F), providing further evidence that lactate is consumed via respiration.

### Kinetic uptake of glucose and lactate *in vivo* show lactate uptake in R3230Ac tumors occurs more rapidly than glucose uptake

**Figure 3:**
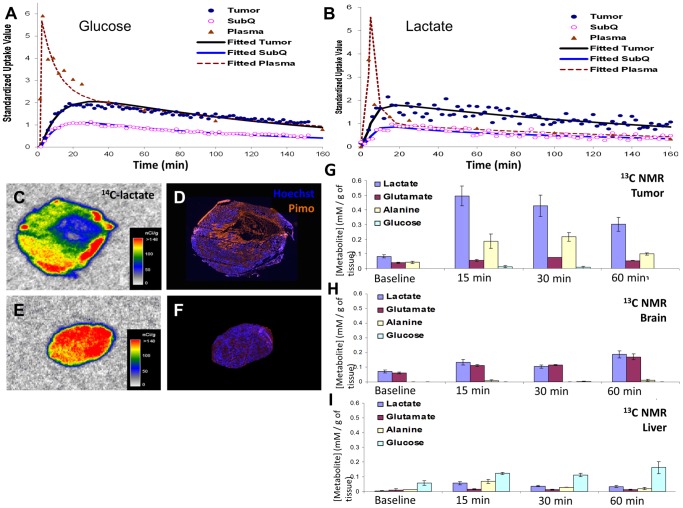
Kinetic analyses of metabolites with radioactive probes show fast plasma clearance of lactate and lactate uptake in perfused regions of R3230Ac tumors. Plots of individual metabolite and fitted pharmacokinetic standard uptake values (SUV) of ^14^C-labeled glucose (100 µCi, A) and lactate (50 µCi, B) infused at a rate of 0.1 mL/min data for plasma, subcutaneous tissue (SQ) and R3230Ac tumor tissue over 160 minutes. R3230Ac tumors, grown in the flanks of Fischer 344 rats, show clearance of ^14^C labeled glucose (n = 6) from plasma from 6 SUV to 2 SUV over 40 mins and maximum uptake of ^14^C labeled glucose in the tumor after 16 mins (A). ^14^C labeled lactate (n = 3) was cleared from the plasma (from 6 SUV to 2 SUV) in 14 mins and showed maximum uptake in the tumor after 14 mins (B). SUV  =  standard uptake rates. Autoradiography images (D-F) of ^14^C-lactate uptake in R3230 Ac tumors show high lactate uptake (C&E) in well-perfused areas, as indicated by positive Hoechst 33342 staining (blue, D&F), compared to hypoxic tumor regions, as indicated by positive pimonidazole staining (orange, D&F). R3230Ac tumors (G) show presence of ^13^C-lactate, ^13^C-alanine and ^13^C-glutamate at 15, 30 and 60 minutes after ^13^C-lactate infusion. All ^13^C metabolites are increased compared to baseline levels (prior to infusion). ^13^C-lactate uptake and ^13^C-metabolite generation in brain (H) and liver (I) after ^13^C-lactate infusion show a slight increase in metabolites compared to baseline but do not reach concentrations found in R3230Ac tumors.

Kinetics of glucose and lactate uptake, retention, and clearance were measured using a novel scintillation probe following i.v. administration of either ^14^C-glucose (n = 9) or ^14^C-lactate (n = 3). A three-compartment pharmacokinetic model was formed from the data to determine rate constants for glucose and lactate uptake by the tumor and subcutaneous (SQ) tissue (Fig S4). Rate constants for ^14^C-glucose and ^14^C-lactate are summarized in Table 2, and the time activity curves were shown in Figure 3A and 3B. Lactate was cleared much faster from plasma than glucose. Furthermore, the rate constant for the transfer of lactate from blood compartment into the tumor was higher than that of glucose (0.238 vs. 0.038), indicating much faster uptake of lactate than glucose. The back flux rate (from tissue back to blood) was higher in lactate compared to that of glucose (0.062 vs. 0.049). As the scintillation probe only detects ^14^C signal, it was not possible to determine if the back flux signal came from free glucose/lactate or their metabolites. The uptake of both glucose and lactate was higher in tumor compared to subcutaneous compartment indicating higher tumor metabolism. The PK analysis shows that R3230Ac tumor tissue takes up lactate faster than glucose.

**Table 2:** Kinetic Transfer Rates for ^14^C-glucose and ^14^C-lactatein R3230Ac Tumors.

K_trans_	Description	*^14^C-glucose*	*^14^C-lactate*	*p*
k_1_	Blood to tumor transfer rate	0.038±0.008	0.238±0.055	<0.001
k_2_	Tumor to blood transfer rate	0.049±0.006	0.062±0.001	0.006
k_3_	Blood to SQ transfer rate	0.0016±0.0001	0.0014±0.0002	0.077
K_4_	SQ to blood transfer rate	0.066±0.008	0.0744±0.009	0.166

Transfer rate constants were calculated from ^14^C-glucose (n = 9) and ^14^C-lactate (n = 3) kinetic data using the compartmental model explained in the Methods and Fig S3. Kinetic transfer rates of ^14^C-glucose were evaluated against the transfer rates of ^14^C-lactate with a two-tailed Student’s T-test.

^14^C-lactate autoradiography was compared in hypoxic (indicated by pimonidazole staining) v. perfused (indicated by Hoechst staining) regions of R3230Ac tumor. We found that the labeled lactate was taken up primarily in well-perfused tumor regions where hypoxia was absent (Fig 3C&D). In one small tumor that did not exhibit appreciable hypoxia, ^14^C-lactate was evenly distributed throughout the whole section (Fig 3E&F). This data is consistent with the proposed metabolic symbiont model [17], in which lactate is preferentially taken-up by better oxygenated tumor regions.

To test if the same metabolites would be generated *in vivo* as *in vitro*, ^13^C-lactate was infused into R3230Ac tumor-bearing rats. Tumor, liver, brain and other organs were collected and snap-frozen at time of sacrifice (15, 30, or 60 min). After 15 minutes, labeled lactate was present in the tumor at 0.5 mM/g of tissue (Fig 3G). Glucose was not significantly produced from lactate. This concentration was lower after 30 and 60 min, indicating metabolism or clearance from the tumor tissue. Both glutamate and alanine concentrations were increased in tumor after lactate infusion, with alanine reaching the highest concentration at 30 minutes (Fig 3G). This shows that the R3230Ac tumor utilizes lactate for metabolite generation. Brain and liver tissue also showed an increase in lactate uptake compared to baseline (Fig 3H&I), but the concentrations seen were not as high as lactate uptake in the tumor. As expected, liver produced glucose from the infused lactate (Cori Cycle).

Taken together, the kinetic results clearly showed: 1. lactate uptake occurs in a rat mammary carcinoma model *in vivo*, 2. Tumor tissue has a higher uptake of lactate than SQ tissue, 3. R3230Ac tumors take up lactate more rapidly than glucose, 4. Lactate uptake occurs in aerobic regions of the tumor, and 5. Glutamate and alanine are generated from lactate *in vivo*. Previous studies have shown that lactate uptake occurs in hepatoma [56], sarcoma [57], and pancreatic cancer [58], and that lactate acts as preferred substrate compared to glucose in gliomas [59], [60]. Here, we have shown for the first time that lactate is also preferentially taken up in a breast cancer model with higher transfer rates than glucose. Further studies with other tumor models would be required to verify whether the preferential uptake of lactate is a class effect in breast cancer. But at the very least, we show that lactate can be taken up and metabolized in vivo, in the presence of glucose.

### Human breast cells metabolize lactate to alanine and glutamate

We questioned if human breast cells could also take up and metabolize lactate. First, we screened the human breast lines (HMEC, MCF7 and MDA-MB-231) for MCT1 and MCT4 expression (Fig S3). MCT1 was expressed in MCF7 and HMEC cells but not MDA-MB-231 cells (Fig S3A), consistent with the literature [18], [26]. MDA-MB-231 was the only cell line that expressed abundant MCT4 (Fig S3B). HMEC showed very little MCT4 expression in the whole cell lysate, and MCT4 was undetectable for MCF7 cells (Fig S3B). Hussien and Brooks provide a more complete characterization of MCT subtype expression in these same cell lines, as they investigated mitochondrial and membrane expression in addition to using whole cell lysates.[18]. Briefly, their studies found MCT1 expression on the plasma membrane of MCF7 and HMEC cells but not MDA-MB-231 cells. MCT4 and MCT2 were found to be expressed in all three cell lines, localizing to the plasma and mitochondrial membranes [18]. Given these differences in MCT subtype expression in cell lines, we hypothesized that lactate metabolism among the breast lines would be different. Specifically, we expected relatively little lactate uptake and metabolism in MDA-MB-231 cells due to their lack of MCT1 expression.

**Figure 4:**
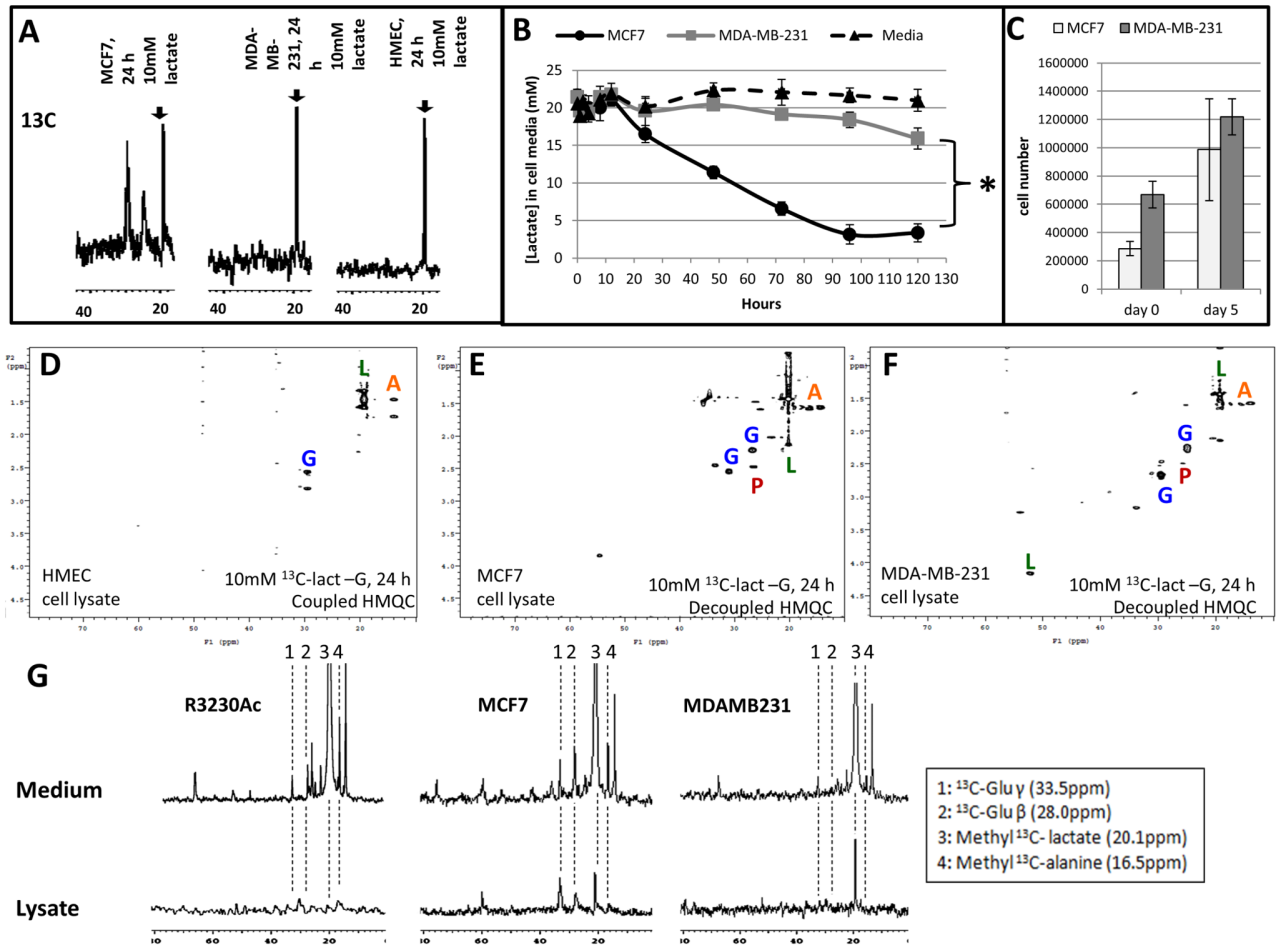
Lactate uptake and metabolism in human breast cells and metabolite excretion. ^13^C NMR spectra of human breast lines indicated evidence of ^13^C-lactate (arrow, 19 ppm) uptake after 24 h exposure to 10 mM ^13^C-3-lactate (**A**). Lactate measurements of cell media after 5 day incubation with 20 mM unlabeled sodium lactate in glucose-free media showed a significant difference in lactate consumption between MCF7 and MDA-MB-231 cells (mean overall decrease in lactate concentrations were 0.4 mM for the no-cell media control plate, 5.5 mM for MDA-MB-231 media and 18.3 mM for MCF7 media, n = 5, *p < 0.001 compared to MDA-MB-231 and media control, Student’s T-test, **B**). The increase in cell number of MCF7 and MDA-MB-231 cells at the beginning (day 0) and end (day 5) of lactate treatment show no significant difference (**C**). Heteronuclear multiple quantum coherence (HMQC) NMR plots of cell lysates treated for 24 h (n = 2) with 10 mM 3-^13^C-lactate (no glucose) showed ^13^C-lactate (dark green “**L**”) uptake and ^13^C-glutamate (blue “**G**”), ^13^C-alanine (orange “**A**”), and ^13^C-pyruvate (red “**P**”) generation in HMEC (**D**), MCF7 (**E**), and MDA-MB-231 cells (**F**). ^13^C NMR spectra of R3230Ac, MCF7 and MDA-MB-231 cell lysates (bottom) and media (top) show evidence of ^13^C-metabolites in the media of each cell line and in the lysate of MCF7 and MDA-MB-231 cells after 24 h incubation with 40 mM lactate (**G**). Numeric labels: 1 = ^13^C-Glu γ, 2 = ^13^C-Glu β, 3 = methyl ^13^C- lactate, 4 = methyl ^13^C-Ala

Both malignant and nonmalignant human breast cell lines showed evidence of intracellular ^13^C-lactate after 24 h treatment (Fig 4A, arrows). This indicates that: 1. lactate uptake occurs in both normal and cancer cells (Fig 4A). 2. lactate uptake is not MCT1-dependent in MDA-MB-231 cells. MDA-MB-231 cells have been documented to express MCT2 [18], which has a higher affinity for lactate than MCT1 [14]. We then sought to compare the rates of lactate uptake between human breast cancer cell lines. We determined lactate concentrations in glucose-free cell media from MCF7 or MDA-MB-231 cells incubated with supplemented lactate (20 mM) over 5 days. The media was not replenished over this time period. Because the lactate used was unlabeled, glucose-free media was chosen so glycolytically-derived lactate would not interfere with the measurement of lactate consumption. Figure 3B illustrates the mean lactate concentration measurements for each time point. Over 5 days, the decline in mean lactate concentrations in the cell media was significantly greater for MCF7 cells (18.3 mM drop) than for MDA-MB-231 cells (5.5 mM drop, p < 0.0001, Fig 4B). When the individual time point measurements of lactate concentration was applied to a linear regression analysis, the rate coefficients of lactate consumption were significantly different between MCF7 (4.01) and MDA-MB-231 (0.71) cells (p < 0.0001). Cell counts at the beginning and end of lactate treatment are displayed in Figure 4C; the increase in cell numbers was not significantly different for MCF7 and MDA-MB-231 cells. However, when cells were plated at equal densities and allowed to grow in high-glucose complete media or treated with 20 mM lactate (no glucose) for 5 days, MCF7 cell numbers showed no difference between treatments. Conversely MDA-MB-231 cells showed a significant increase in cell numbers when cultured in the glucose-containing lactate-supplemented media (Fig S5). These results show that MCF7 cells utilize lactate more effectively than MDA-MB-231 cells and have less of a dependence on glucose for cell growth than MDA-MB-231 cells. These results reinforce the findings in our previous study [17] that cell lines with high MCT1/low MCT4 consume more lactate than cell lines expressing high MCT4/low MCT1.

HMQC plots generated from human breast cell lysates showed evidence of ^13^C-labeled alanine, glutamate and pyruvate in human normal breast cell lysates (Fig 4D) and human breast cancer cell lysates (Fig 4E&F) after 24 h incubation with 10 mM 3-^13^C-lactate, indicating that lactate can be catabolized in these cell lines and that the metabolites generated are the same as those seen in R3203 Ac cells. Taken together, these data show that MCF7 and MDA-MB-231 cells consume lactate at significantly different rates but produce similar catabolites. Our original hypothesis that normal breast and breast cancer cells would metabolize lactate differently was incorrect, as HMEC cells showed the same metabolites generated from exogenous lactate as breast cancer cell lines; however, the relative rates of lactate utilization and catabolism for each cell line are different.

### Lactate-derived metabolites are exported from cells

Since lactate was not toxic to cells in the physiologically relevant range, we hypothesized that lactate-generated metabolites could be released from cells after 24 h as a means to reduce levels within the cells. Media and cell lysates from each breast cancer cell line were collected after 24 h treatment with 5 mM ^13^C-lactate (-glucose). Each of the cell lines showed ^13^C-labeled alanine and glutamate in the media (Fig 4G). R3230Ac cells showed very little lactate, alanine and glutamate in cell lysates after 24 h, indicating that lactate metabolism is more rapid in these cells compared to MCF7 or MDA-MB-231 cells, which both retained some ^13^C-lactate in the lysate(Fig 4G). Additionally, there is evidence of a greater amount of glutamate (peaks “1” and “2”) in both the R3230Ac and MCF7 cells compared to MDA-MB-231 cells (Fig 4G). Although lack of MCT1 expression in MDA-MB-231 cells did not prevent lactate uptake, the lactate catabolism of these cells was considerably less efficient than the R3230Ac or MCF7 cells. Studies in normal cell lines (HMEC and HUVEC) also showed lactate uptake and metabolite release into the media after treatment with 10 mM lactate for 4.5 h (HMEC, Fig S6A&B) or 5 mM lactate for 24 h (HUVEC, Fig S6C). These results show that excretion of lactate catabolites appears to be a universal trait, shared between normal and tumor cells.

### CHC prevents lactate uptake at lactate concentrations ≤20 mM; CHC prevents lactate catabolism at lactate concentrations of 40 mM

Alpha-cyano-4-hydroxycinnamate (CHC) is a small molecule inhibitor of MCTs, with a ten-fold selectivity for MCT1 compared to MCT4 [61]. Previously, we have shown that 5 mM CHC will inhibit lactate uptake, decrease cellular ATP in SiHa and WiDr cells and decrease tumorigenicity [17]; however, this study was not conducted with breast cancer cell lines, and 5 mM CHC is a rather high concentration [62]. It has been reported that concentrations of CHC between 50-500 µM will inhibit MCT1, and that the mitochondrial pyruvate carrier (MPC) is inhibited with concentrations ≤ 5 µM [14], [63]. To span the range of pharmacologically effective doses, we tested the effects of 5 and 0.1 mM CHC on cell viability and lactate metabolism. The concentration of 5 mM CHC was included based on previous studies [17], [64]. The lower concentration (0.1 mM) was chosen based on previous commentary [62] and evidence that 5 mM CHC may elicit nonspecific cell death responses [65].

**Figure 5:**
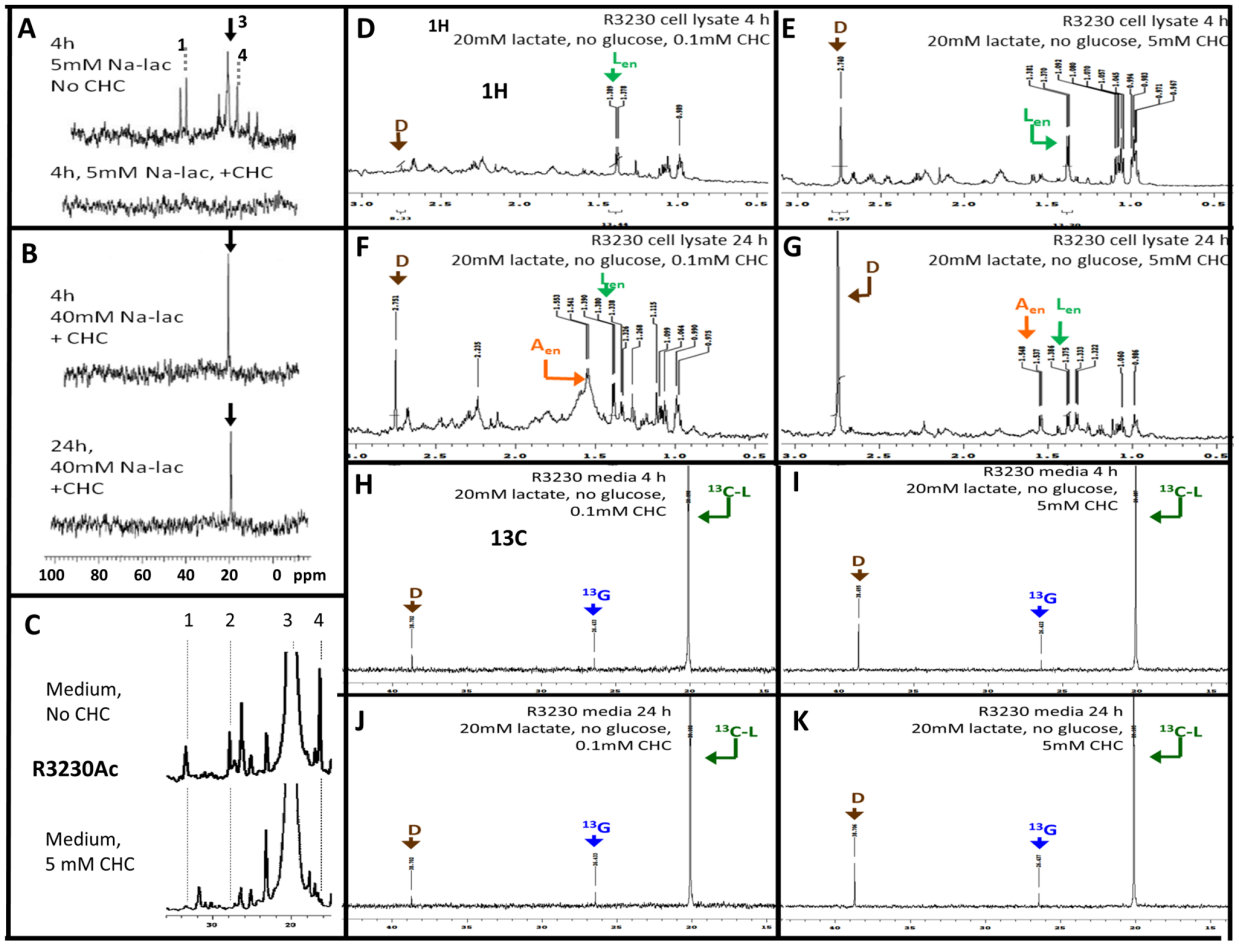
Inhibition of exogenous lactate uptake and endogenous lactate excretion with addition of CHC. All experiments represented were carried out in glucose-deprived conditions. ^13^C spectra of R3230Ac cell lysates incubated for 4 h with 5 mM ^13^C-lactate without CHC treatment shows peaks corresponding to lactate (“3”), alanine (“4”) and glutamate (“1”) (**A**, top). ^13^C spectra of R3230Ac cell lysates incubated for 4 h with 5 mM ^13^C-lactate with 5 mM CHC treatment show no peaks corresponding to lactate or metabolites (**A**, bottom). ^13^C spectra of R3230Ac cell lysates incubated for 4 h and 24 h with 40 mM ^13^C-lactate + 5 mM CHC shows a peak corresponding to lactate (arrow) but no other metabolites (**B**). ^13^C spectra of R3230Ac cell media incubated with 5 mM ^13^C-lactate for 4 h shows peaks corresponding to alanine and glutamate without CHC treatment (**C**, top); metabolite peaks are absent or smaller with 5 mM CHC (**C**, bottom). ^1^H spectra of R3230Ac cell lysate with the following treatments: 20 mM ^13^C-lactate + 0.1 mM CHC for 4 h (**D**) 20 mM ^13^C-lactate + 5 mM CHC for 4 h (**E**), 20 mM ^13^C-lactate + 0.1 mM CHC for 24 h (**F**), 20 mM ^13^C-lactate + 5 mM CHC for 24 h (**G**). ^13^C spectra of R3230Ac cell media with the following treatments: 20 mM ^13^C-lactate + 0.1 mM CHC for 4 h (**H**) 20 mM ^13^C-lactate + 5 mM CHC for 4 h (**I**), 20 mM ^13^C-lactate + 0.1 mM CHC for 24 h (**J**), 20 mM ^13^C-lactate + 5 mM CHC for 24 h (**K**).Endogenous lactate  =  green “**L_en_**”, endogenous alanine  =  orange “**A_en_**”, ^13^C-lactate  =  dark green “**^13^C-L**”, ^13^C-glutamate  =  blue “**^13^G**”, DMSO  =  brown “**D**”

Using ^13^C-lactate (-glucose), we investigated the ability of 5 mM CHC to prevent lactate uptake with low (5 mM) and high (40 mM) lactate concentrations (Fig 5A-C). Cell lysates from R3230Ac cells show metabolism of 5 mM ^13^C-lactate to alanine and glutamate after 4 h in the absence of CHC (Fig 5A, top). When 5 mM CHC was added to the cell media with 5 mM ^13^C-lactate (-glucose), the cell lysate did not show evidence of ^13^C-lactate after 4 h (Fig 5A, bottom). When R3230Ac cells were exposed to 40 mM ^13^C-lactate and 5 mM CHC for 4 h, cell lysates showed a large ^13^C-lactate peak in the ^13^C spectrum (Fig 5B, top), and ^13^C-lactate was still present after 24 h (Fig 5B, bottom). From previous observations, it is anticipated that all lactate metabolites should be exported at this time point (Fig 4G). The fact that a rather large ^13^C-lactate peak was still detected after 24 h indicates that treatment with 5 mM CHC may be more effective at preventing lactate exportation than inhibiting uptake when extracellular lactate concentrations were high. Because CHC is a competitive inhibitor of MCT1, it is possible that lactate enters the cell when extracellular concentrations are high due to lactate outcompeting CHC. It should be noted that no ^13^C-metabolites were present in the R3230Ac cell media treated with 40 mM ^13^C-lactate + 5 mM CHC, indicating that 5 mM CHC prevented lactate catabolism even though it was taken up. Furthermore, with addition of 5 mM CHC, there were less ^13^C-labeled metabolites present in the media of R3230Ac cells treated with 5 mM ^13^C-lactate for 4 h, as expected with inhibition of lactate uptake at this lactate concentration (Fig 5C).

We next investigated the effects of low (0.1 mM) v. high (5 mM) CHC concentrations on lactate metabolism at a mid-range concentration (20 mM). The ^13^C spectrum of the media only (blank) did not show evidence of large metabolite peaks, indicating that background noise was low (Fig S7A). Previously, we have shown that this concentration of ^13^C-lactate will be taken up by R3230Ac cells (Fig 2B). In the ^1^H and ^13^C spectra of R3230Ac cells treated with low (Fig S7B&D) or high (Fig S7C&E) CHC in the absence of ^13^C-lactate, no ^13^C-lactate peaks were evident. Panels D-G in Figure 9 show ^1^H spectra for R3230Ac cells after 4 h (Fig 5D&E) or 24 h (Fig 5F&G) exposure to 20 mM ^13^C-lactate (-glucose) plus either 0.1 mM CHC (Fig 5D&F) or 5 mM CHC (Fig 5E&G). No ^13^C-lactate was evident in the ^1^H spectra of R3230Ac cell lysates after 4 h or 24 h incubation with 20 mM ^13^C-lactate with low or high CHC (Fig 5D-G). Additionally, there was no evidence of ^13^C-metabolites in any of the cell lysates (Fig 5D-G). We could not acquire ^13^C spectra on these samples due to the low signal. Endogenous lactate (“L_en_”) was present in all cell lysates (Fig 5D-G). Glucose was not included in the media; therefore, glucose could not be the source of the endogenous lactate. Formation of endogenous lactate was derived from some other metabolite present, most likely glutamine or glycogen, as there have been reports of tumor cells with high glycogen content [66]-[70]. The presence of the endogenous lactate peak was evident after both 4 h and 24 h incubation, indicating that both low and high CHC partially inhibited endogenous lactate excretion (Fig 5D-G). Endogenous alanine (“A_en_”), which was formed from the endogenous lactate, was present in cell lysates after 24 h regardless of the presence of high or low CHC (Fig 5F&G).

In addition to R3230Ac cell lysates, we collected cell media from these experiments (Fig 5H-K). In each of the ^13^C spectra, an abundance of ^13^C-lactate was seen in the cell media, indicating that both high and low CHC concentrations effectively inhibited a majority of ^13^C-lactate uptake. Peak heights are very similar regardless of incubation time or CHC concentration; 0.1 mM CHC was as effective as 5 mM CHC for inhibiting lactate uptake. There was evidence of ^13^C-glutamate generation (verified by HMQC, data not shown) and exportation into the media for each sample (Fig 5H-K). In the ^1^H spectra of R3230Ac cell lysates, a large ^13^C-glutamate peak was not apparent after 4 h or 24 h; however, there was evidence of a peak ∼2.23 ppm, which corresponds to ^13^C-glutamate (Fig 5D-G). This indicates that the small amount ^13^C-lactate that was taken up in the presence of CHC was converted to ^13^C-glutamate before the 4 h time point. This would suggest that pyruvate movement into mitochondria by the MPC was not completely inhibited by 5 mM CHC. The cell lysate samples do not have resolution comparable to the ^13^C spectra, which is why the ^13^C-glutamate peak is evident in the ^13^C spectra of the media but not as apparent in the ^1^H spectra of the cell lysates. Furthermore, the ^1^H spectra of the R3230Ac cell media showed the presence of endogenous lactate in the media at relatively similar quantities (Fig S8A-D), which implies that either concentration of CHC provided incomplete inhibition of endogenous lactate exportation. The small peaks in the ^1^H spectra of the cell media that may represent ^13^C-glutamate are not much higher than background levels, which is why we generated ^13^C plots (Fig 5H-K).

### 5 mM CHC significantly increased cell death in MCF7 and R3230Ac cell lines independent of the presence of exogenous lactate

MCT1 inhibitors have been given some attention as putative anti-cancer therapies [16], [17], [20]. We sought to characterize cell viability and death responses in MCF7 and R3230Ac cells to high (5 mM) or low (0.1 mM) CHC in the presence or absence of glucose ± high lactate (40 mM). If both the lower and higher CHC concentration showed significant cell death when lactate was supplemented, then lactate toxicity would be due to the inability of the cell to “detoxify” the lactate via biochemical pathways (generation of alanine and glutamate) or due to factors associated with prevention of lactate excretion. Of note, it has previously been shown that cell death is elicited by other MCT1 inhibitors via the inability of the cell to effectively regulate the lower pH_i_ that results from lactic acid accumulation from glycolysis [20]. If, however, cell death was elicited by another mechanism with high CHC, we would expect that the lower CHC concentration would not show any cell death. MCF7 cells were chosen as our human breast cancer model; MDA-MB-231 cells were deemed unfit for these experiments due to their lack of MCT1 expression. R3230Ac cells were also used for these studies to maintain consistency with our *in vivo* model and to test effects of CHC on an avid lactate-consuming cell line.

**Figure 6:**
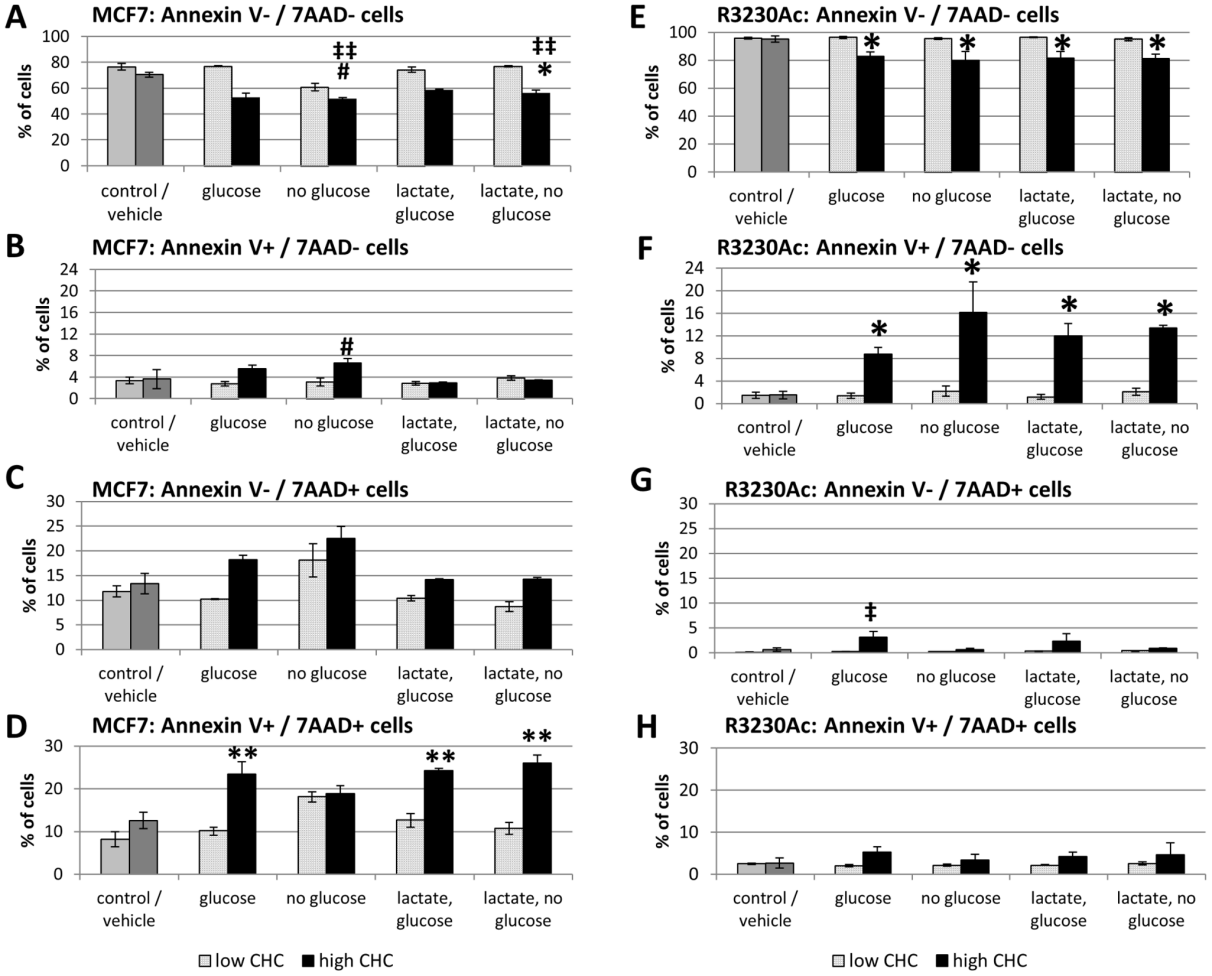
24 h exposure to 5 Cell viability as measured by Annexin V –/7-AAD – labeling (n = 3) in MCF and R3230Ac cells treated with high (5 mM) or low (0.1 mM) CHC with and without glucose (no lactate) and with 40 mM lactate (with and without glucose). Viable MCF7 cells show significant decreases with 5 mM CHC (A, #p≤0.004 compared to control (no CHC, no lactate + glucose), ‡‡p < 0.01 compared to 40 mM lactate-treated MCF7 cells, *p = 0.003 compared to all no CHC and low CHC groups). Percentage of apoptotic (Annexin V+/7-AAD-) MCF7 cells show significant increases with the –glucose-lactate+high CHC treatment and the +glucose+lactate+high CHC treatment (#p < 0.05) and between the –glucose–lactate+high CHC group and 40 mM lactate treatments without CHC (+ or – glucose) (†p < 0.05, B). Percentage of MCF7 cells with loss of membrane integrity (Annexin V–/7-AAD+) show no significant differences with CHC treatment (C). Percentage of MCF7 cells marked for both cell death pathways (Annexin V+/7-AAD+) show significant increases with 5 mM CHC compared to the no CHC and low CHC groups (**p < 0.05, D). Percentage of viable R3230Ac cells show significant decreases with 5 mM CHC compared all no CHC and low CHC groups (*p < 0.008, E). Percentage of apoptotic (Annexin V+/7-AAD-) R3230Ac cells show significant increases with 5 mM CHC compared to compared to no CHC and low CHC groups (*p < 0.02, F). Percentage of R3230Ac cells marked for loss of membrane integrity (Annexin V–/7-AAD+) show no significant differences except–glucose-lactate+high CHC treatment (‡p < 0.05, G). Percentage of R3230Ac cells marked for both cell death pathways (Annexin V+/7-AAD+) show no significant changes with any treatment (H). Results analyzed with One-Way ANOVA and Bonferroni/Dunn post-hoc tests.

Fig 6A&E shows the percent of total MCF7 and R3230Ac cells that were viable (Annexin V–/7AAD-) after treatment with high (5 mM) or low (0.1 mM) CHC concentrations with or without glucose and/or 40 mM lactate. The control group represents cells in complete media without lactate or CHC treatment. In MCF7 cells, high CHC + glucose did not significantly change viability. Without glucose in the media, the percentage of viable cells dropped from 80% to 56% (#p≤0.004 compared to control, Fig 6A). This decrease in viable cells was significant compared to MCF7 cells treated with 40 mM lactate + glucose (‡‡p < 0.01, Fig S3B). High CHC + high lactate and no glucose resulted in 59% viability. This result was significant compared (*p = 0.003, Fig 6A) to all groups with no or low CHC, except for the no glucose, no lactate low CHC group. When a linear regression model was conducted for MCF7 cells, it was found that CHC concentration and glucose availability significantly influenced cell viability (p < 0.0001 and p = 0.001, respectively) but the presence of high lactate showed no significant changes in cell viability. All high CHC treatments in R3230Ac cells showed a significantly decreased percentage of live cells compared to all groups with no or low CHC (*p < 0.008; Fig 6E). These results show that cell viability with this assay is influenced more by CHC concentration and availability of glucose than the presence of lactate.

Also available from the data set is whether cells died via an apoptotic pathway (Annexin V+/7-AAD-), loss of membrane integrity (Annexin V–/7-AAD+) or marked for both (Annexin V+/7-AAD+) after CHC treatment. Apoptosis was significantly increased with high CHC in absence of glucose or lactate, but with those nutrients added back alone or together cells were protected from apoptosis (#p < 0.05; Fig 6B). R3230Ac cells showed a significant increase in the percentage of apoptotic cells with high CHC treatment compared to all no and low CHC treatments (*p < 0.02, Fig 6F).None of the treatments affected the loss of membrane integrity of MCF7 or R3230Ac cells (Fig 6C). The percentage of MCF7 cells marked for both apoptosis and loss of membrane integrity was significantly increased with all high CHC treatments (except the high CHC –glucose, -lactate group) compared to all no or low CHC groups (**p < 0.05, Fig 6D). There were no significant changes in the percentage of R3230Ac cells marked for both apoptosis and loss of membrane integrity in any treatment (Fig 6H).

These results show that R3230Ac cells primarily undergo apoptosis in response to high CHC and that MCF7 cells stain positive for both cell death pathways in response to high CHC. In all the statistical tests conducted, low CHC concentration was not significantly different from the untreated control or vehicle alone. The presence or absence of lactate showed no significance in the statistical models. In summary, the cell death responses elicited from 5 mM CHC are independent of the presence of exogenous lactate; therefore, our original hypothesis that CHC would elicit cell death in lactate-treated, glucose-deprived cells due to the inhibition of lactate uptake and utilization was incorrect.

### Exogenous lactate is taken up but not catabolized in hypoxic R3230Ac cells

**Figure 7:**
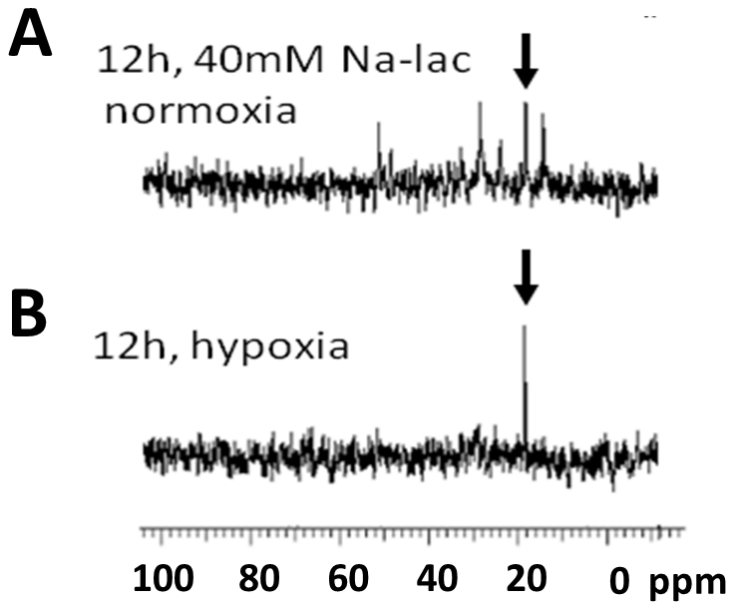
Hypoxic R3230Ac cells take up ^13^C-lactate. Treatment with 40^13^C-lactate in glucose-deprived, normoxic R3230Ac cancer cells result in lactate uptake and metabolism after 12 h (**A**). Under hypoxic conditions, evidence of lactate uptake (arrow) but no additional labeled metabolites can be seen in the cell lysates after 12 h (**B**). Arrow: ^13^C-methyl lactate.

Figure 7C&D showed that lactate uptake occurs primarily in aerobic regions of R3230Ac tumors. It is known that hypoxic cells upregulate glycolysis, resulting in higher lactate production [71]. This glycolytically-derived lactate will then be exported from the cell, resulting in high lactate accumulation in hypoxic areas of the tumor [10]. Therefore we hypothesized that hypoxic cells would take up exogenous lactate, but would not be able to catabolize it. The ^13^C spectra of glucose-deprived, ^13^C-lactate-treated R3230Ac cells show uptake and utilization of ^13^C-lactate, with many labeled metabolites present after 12 h. In glucose-deprived and hypoxic conditions, after 12 h incubation with 40 mM ^13^C-lactate, the ^13^C spectra of R3230Ac cell lysates show evidence of the lactate peak but no corresponding labeled metabolites. This indicates that although hypoxic cells can take up lactate, it is cannot be utilized for metabolite generation.

## Discussion

Our study shows that breast cancer cells tolerated and catabolized lactate at concentrations found in human breast cancer. Breast cancer cell lines showed different rates of lactate uptake and generation of similar catabolites (glutamate and alanine) *in vitro* and *in vivo*. *In vivo*, uptake of lactate occurred more quickly than uptake of glucose in tumor tissue, and this uptake coincided with perfused (rather than hypoxic areas) of the tumor. *In vitro*, hypoxic breast cancer cells did not show catabolism of lactate. Taken together, our study strongly supports one side of the metabolic symbiont model: the assertion that aerobic breast cancer cells can tolerate and metabolize lactate. Our autoradiography data provide further evidence that the metabolic symbiont model may operate in some solid tumors. Manipulation of lactate uptake and catabolism was accomplished with use of CHC, but higher concentrations of CHC killed breast cancer cells in a manner that was not dependent upon the presence of lactate. This suggests that cytotoxic activity of CHC is unpaired from lactate metabolism in our model.

In the past we have shown that lactate can be used as a substrate for aerobic cancer cells and that MCT1 and MCT4 expression correlate with the ability of certain cancer cell lines to consume lactate [17]. Here, we showed that cellular responses to exogenous lactate varied depending upon cell type and glucose availability, but, overall, at concentrations seen in human breast cancer, lactate was well-tolerated (Figs S1 & S2) and catabolized (Figs 3–5). Cell lines that consume more lactate ( “high lactate-consumers”) showed less of a reliance on glucose for cell growth compared to cell lines that consumed less lactate (“low lactate-consumers”) (Fig 5, S5, 8). The R3230Ac tumors demonstrated a significantly higher kinetic uptake rate for lactate than for glucose (Fig 3) and showed no changes in cell survival with glucose-deprivation (Fig S1D & S2D), providing additional evidence for a high lactate-consuming phenotype.

In all of the experiments conducted, the concentration of glutamine was not altered, and therefore present at concentrations of the manufacturer’s supplementation (4 mM). Glutamine has been shown to be an important metabolite for growth of cancer cell lines in culture [67]; therefore, we chose to not remove it. Previous reports indicate that the contribution of glutamine to lactate formation is approximately 7–13%, depending on the growth phase and other metabolites in the culture media [66]. While glutamine may contribute to a small amount of lactate formation [66], the major metabolite responsible for lactate formation is glucose. The significance of convergent pathways of glutamine and lactate metabolism may warrant future study, especially in tumor cells that may demonstrate less of a dependence on glucose as a primary substrate.

**Figure 8:**
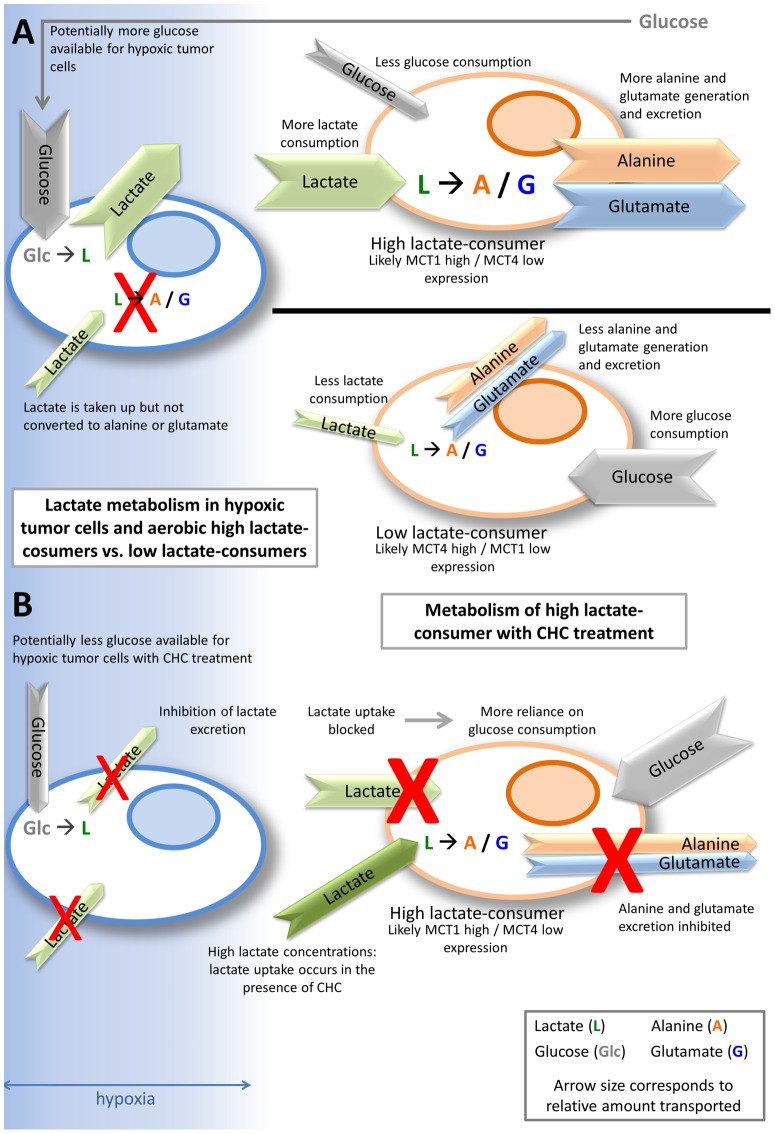
Summary diagram of lactate metabolism in high lactate-consumers vs. low lactate-consumers. The blue gradient represents oxygen diffusion. The cell on the left is hypoxic; the cells on the right are aerobic. Arrow colors correspond to substrates, and arrow size corresponds to relative amount. Hypoxic tumor cells take up glucose (gray “**Glc**”) and produce lactate (dark green “**L**”), leading to higher concentrations of lactate. Lactate may be taken up by the hypoxic tumor cells, but it is not catabolized. Aerobic tumor cells that are high lactate-consumers and likely express high MCT1/low MCT4 can take up lactate and catabolize it to alanine (orange “**A**”) and glutamate (blue “**G**”), which will be exported from the cell. With the aerobic high lactate-consumer cell consuming lactate, more glucose can potentially be spared for hypoxic tumor cell use, potentially conferring a survival advantage. Aerobic tumor cells that are low lactate-consumers and likely express low MCT1/high MCT4 take up less lactate than the high lactate-consumers, consequently producing and exporting less alanine and glutamate. Low lactate-consumers utilize more glucose, which will not allow glucose to reach the hypoxic tumor cells (**A**). One proposed strategy for starving hypoxic tumor cells of glucose in a high lactate-consuming tumor is to treat with a MCT1- inhibitor, like CHC. CHC prevents lactate uptake and catabolism in cells, forcing the aerobic high lactate-consumer to use glucose, which starves the hypoxic tumor cell of glucose. Lactate transport out of hypoxic cells is also inhibited, which would also lead to hypoxic cell death (**B**).

For lactate to act as an energy substrate, it needs to be converted to pyruvate, enter the mitochondria and go through oxidative phosphorylation. Through use of labeled lactate, it has been previously found that lactate completes the course of oxidative phosphorylation, as shown by generation of labeled CO_2_ [56], [72]. Likewise, we found that R3230Ac cells significantly increased oxygen consumption with increasing lactate concentrations (Fig 2E). Another strong indication of the use of labeled lactate as a substrate to enter the TCA cycle that we found in our study was the appearance of labeled glutamate in the cell lysates and media of all cell lines tested (Figs 5–8). The most straight-forward pathway for ^13^C-glutamate formation is: ^13^C-L-lactate ↔ ^13^C-pyruvate → → TCA cycle partial completion → ^13^C- α –ketoglutarate → ^13^C-glutamate. We also identified alanine peaks after adminstration of ^13^C-L-lactate. Labeled alanine formation occurs through: ^13^C-L-lactate ↔ ^13^C-pyruvate + glutamate ↔ ^13^C-alanine + α-ketoglutarate. Alanine and glutamate (whether labeled or endogenous) can participate in the reaction catalyzed by alanine aminotransferase (α -ketoglutarate + alanine ↔glutamate + pyruvate). While this seems to only shift the balance of lactate catabolites, this reaction may serve to temporarily alleviate nitrogen stress in the cell by transferring it to glutamate. When alanine is formed in other organs, particularly in skeletal muscle, it can be shuttled to the liver to participate in the Alanine Cycle (similar to the Cori Cycle) [73]. Alanine generated in the tumor is unlikely to participate in the Alanine Cycle, as the diffusion of metabolites to a blood supply and transport is limited by vessel arrangement and efficiency, respectively [74]. The alanine generated may be used for anabolic purposes by the tumor, but it is also possible that alanine participates in futile metabolic cycles. The metabolic fate of these lactate catabolites would be an interesting avenue of further research, and future studies could determine whether inhibiting lactate catabolism pathways in tumors may provide another therapeutic option.

No prior reports have compared the consumption of lactate to that of glucose *in vivo*. We showed that the R3230Ac tumor consumes glucose and lactate simultaneously *in vivo*, converting lactate to alanine and glutamate. Our kinetic data support avid uptake of ^14^C-lactate by the tumor *in vivo*, with uptake rate constants that are higher than that of glucose. These results suggest that lactate may be an important substrate for the R3230Ac tumor line. Second, we demonstrated that lactate was taken-up most preferentially in oxygenated tumor regions (Fig 4). In prior studies, we demonstrated that lactate accumulation was highest in perinecrotic hypoxic tumor regions of the R3230Ac tumor [10]. These results strongly suggest that at least part of the lactate that is produced by hypoxic tumor regions may diffuse down its own concentration gradient, toward better perfused aerobic cells, which can then take it up via the MCT transporters and utilize it. In other work by our group we have indeed found this to be the case [17], which leads to preferential death of hypoxic tumor cells, substantial growth delay and increased radiation response. Our *in vivo* results support this suggestion that there may be a cooperative relationship between different cells within the R3230Ac tumor. Recently, it has been reported that lactate uptake in endothelial cells contributes to increased angiogenesis and HIF-1α expression [64], [75]. The ability of tumor stroma to consume lactate supports tumor growth. Additionally, other groups have shown that tumor-associated fibroblasts undergo aerobic glycolysis, producing lactate, which can then be utilized by tumor cells; this has been termed the “Reverse Warburg Effect” [76]–[79]. *In vitro*, we observed production of both alanine and glutamate in R3230Ac cells as well as normal human breast epithelial cells and human breast cancer cell lines after administration of ^13^C-lactate. The fact that lactate utilization was seen in human breast cancer as well warrants further investigation of their lactate-consuming ability *in vivo,* as the lactate-consuming phenotype of particular tumors may influence treatment strategies.

Therapeutically targeting lactate metabolism in tumor cells has been proposed [16], [17], [64], [80] and is currently being evaluated in Phase I/II clinical trials [81]. Previously, inhibition of MCT1, specifically with CHC, was used to block lactate uptake in more oxidative cells, thereby starving their more hypoxic neighbors of glucose [17]. While these results are encouraging, it is well documented that CHC does not only inhibit lactate transport. MCTs transport many monocarboxylates. Though each subtype has a different affinity for particular monocarboxylates, the most widely-expressed and well-characterized subtypes (MCT1, 2 and 4) are capable of transporting lactate, pyruvate, butyrate and ketone bodies [14]. Inhibiting the transporter will theoretically prevent trafficking of each of these monocarboxylates. Diers *et al.* recently reported that 0.5 mM CHC treatment prevented pyruvate uptake and inhibited mitochondrial respiration in breast cancer cells [82]. At the CHC concentrations used in our study (0.1 and 5 mM), it is likely that pyruvate (in addition to lactate) transport was also impaired.

Despite the ability of 5 mM CHC to inhibit lactate catabolism and export at 40 mM concentrations (Fig 5B), we found that 40 mM lactate was not cytotoxic to MC7 or R3230Ac cells (Fig 6). High CHC elicited significant cell death independent of addition of exogenous lactate, indicating that off-target effects are responsible for cancer cell death at this concentration of CHC. These results suggest that CHC may not be the best therapeutic MCT1 inhibitor. Other MCT1 inhibitors have elicited cancer cell death via decreased pH_i_ with treatment [20]. It is important to also address the potential toxicity with MCT1 inhibition in normal tissue as well as in other diseases that show high lactate levels, such as meningitis or sepsis [83], [84]. Encouragingly, in a recent study, we found that mice treated with CHC showed no morbidity [85].

Targeting tumor metabolism has been proposed as an anti-cancer therapy, specifically, MCT1 inhibition has received attention in recent years [16], [17], [20], [64], [80], [81]. Our study supports the rationale that it is important to first know the metabolic phenotype of the individual tumor before administering metabolic intervention. With all breast cell lines tested, we saw tolerance of lactate at concentrations relevant to breast cancer. Our results demonstrate two different lactate-consuming phenotypes: high lactate-consumers (R3230Ac and MCF7) and low lactate-consumers (MDA-MB-231) (Fig 8). We showed that lactate consumption and glucose dependence differ between the lactate-consuming phenotypes and that catabolite generation from extracellular lactate was not equal in relative rates or amounts, with low lactate-consumers (MDA-MB-231 cells) showing smaller peaks of alanine and glutamate compared to high lactate-consumers (R3230Ac or MCF7 cells) (Fig 8). Inhibiting lactate uptake in aerobic R3230Ac cells may starve their hypoxic neighbors (metabolic symbiont); however, this model needs to be studied in more detail *in vivo* to assess its overall influence on tumor survival. Our studies also indicate that tumors that behave similar to MDA-MB-231 cells (lacking MCT1 expression and/or showing low lactate consumption) may not be a good candidate for manipulation of lactate pathways. Given these inherent differences, a single approach for metabolism manipulation is not appropriate; therapeutically targeting tumor metabolism would need to be tailored to particular metabolic phenotypes.

In clinical studies, it has been found that lactate accumulation is an indicator of shorter metastasis-free and overall patient survival [2], [34], [35], [38], [39], [46]. A recent study measuring lactate accumulation and spatial distribution in prostate cancer compared the aggressive, anaplastic, fast-growing Dunning R3327-AT to the parental, well-differentiated, slow-growing Dunning R3327-H in animal models [86]. Similar to the findings in human solid tumors, the more aggressive AT tumor line showed significantly more lactate accumulation and necrosis, specifically in the tumor core [86]. Not surprisingly, the same conclusion from human and animal studies emerges: lactate accumulation is a reliable indicator of tumor aggressiveness, associated with fast growth and necrosis within a solid tumor [10], [22], [35], [38], [39], [47], [86]. The topic of lactate utilization is investigated comparatively less, and the importance of the lactate-consuming phenotype is currently unknown. Our study has investigated lactate uptake and catabolism in three breast cancer cell lines and two normal cell lines *in vitro* and in one breast tumor model *in vivo*. Comparing lactate consumption in benign and malignant tumor models is the next step in elucidating the importance of lactate consumption to tumor survival and/or aggressiveness.

Lactate accumulation and utilization are two sides of the same coin. The unanswered questions regarding the ability of the tumor to utilize lactate are: 1. Is it an indicator of a less aggressive tumor or is the ability to consume lactate a survival advantage? (Are these questions mutually exclusive?) 2. Can we effectively alter (and sustain the alteration of) the lactate-consuming phenotype of a tumor through manipulation of metabolic pathways? Recently there has been increasing interest in targeting lactate metabolism in tumors. A greater understanding of the complex and dynamic metabolic pathways that operate in tumors provide more avenues for tailored treatments.

## Methods

### Lactate, CHC and Cell Culture

Sodium L-lactate (C_3_H_5_NaO_3_) was purchased from Sigma-Aldrich (St. Louis, MO, USA). All sodium lactate concentrations for cell treatments were made by diluting the powder in appropriate cell media. Solutions were discarded after 2 weeks. For NMR, sodium L-lacate-3-^13^C solution (45–55% w/w in H_2_O) 99 atom % ^13^C was acquired from Isotec (Sigma, St. Louis, MO). Alpha-cyano-4-hydroxy-cinnamate (CHC) powder was acquired from Sigma (St. Louis, MO) and dissolved in DMSO to create 1 M and 100 mM stock solutions. The stock solution was passed through a 0.22 µm filter prior to dilution in treatment media to acquire concentrations equal to 0.1, 1, 2.5, 4, or 5 mM.

Cell lines used include MCF7 (ER+ human breast adenocarcinoma), MDA-MB-231 (triple negative human breast adenocarcinoma), HMEC (human mammary epithelial cells), and R3230Ac (rat mammary carcinoma. Cells were maintained in 37°C, 5%CO_2_, 20% O_2_ in a Forma Scientific (Marietta, OH) incubator. All cell lines were acquired through Duke University’s Cell Culture Facility and from ATCC (Manassas, VA), except HMEC, which were acquired from Clonetics (Switzerland). HMEC cells were cultured in MEBM media from Lonza/Clonetics (Switzerland) with added supplements (“MEGM media”, Single®Quots®: 2 mL BPE, 0.5 mL hEGF, 0.5 mL insulin, 0.5 mL hydrocortisone, 0.5 mL GA-1000). All other cell lines were cultured high glucose DMEM (Gibco) +10% FBS + 1% antibiotic/antimycotic. For glucose deprivation experiments, DMEM without glucose or sodium pyruvate was used (Gibco) +10% FBS + 1% antibiotic/antimycotic. In all experiments, L-glutamine was present in DMEM media at the manufacturer’s concentration; glutamine was never omitted or altered. Hypoxia experiments were carried out in the Invivo_2_ 500 hypoxia chamber (Ruskinn Technology Ltd, Pencoed, Bridgend, UK). Gas mixtures were 0.5% O_2_, 5% CO_2_, and 94.5% N_2_.

### *In vitro* sodium lactate measurements in cell media

Unlabeled sodium lactate was measured in cell media with the commercially available first-generation (discontinued in favor of the second-generation) Lactate Pro measuring device, which has a reliable measuring range between 0.8 – 23 mM. Cells were plated at a known density and allowed to reach 60% confluency before the beginning of the experiment. At this time, the cell media was changed from high glucose DMEM (+ 10% FBS) to glucose-free DMEM (+ 10% FBS). Time zero was designated at the addition of 20 mM exogenous lactate. Lactate measurements were taken at times 0, 1, 2, 4, 8, 12, 24, 48, 72, 96 and 120 h. The media was not changed over the entire 5-day period. Lactate measurements were conducted as follows: 100 µL of cell media was placed on the Lactate Pro measuring strips that were then inserted into the Lactate Pro device. A standard curve of known lactate concentrations was performed, and the device was calibrated with a known lactate concentration prior to experimental measurements (SEM  =  ± 0.34 mM). At the end of the experiment, cells were counted. Five independent experiments were conducted. Overall mean lactate concentration decreases in the cell media and the amount of lactate consumed per cell were calculated. A linear regression analysis was conducted on the lactate measurements. Rate coefficients were determined after controlling for the time and batch effects.

### Cell Growth in vitro in lactate media vs. glucose media

MCF7 and MDA-MB-231 cells were plated at equal densities and allowed to reach 60% confluency before the beginning of the experiment. At this time, the cell media was changed from high glucose DMEM (+ 10% FBS) to either glucose-free DMEM (+ 10% FBS) with supplemented 20 mM lactate or high-glucose DMEM (+10% FBS). Cells were allowed to grow for 5 days before being harvested and counted.

### Cryosectioning, Bioluminescence and Image analysis

Collection of human tissue LABC biopsies was approved by Duke University Health System Institutional Review Board (IRB), and patient samples were de-identified before acquisition for cryosectioning. All patients provided written informed consent to participate in the study. The Duke University Institutional Review Board approved the protocol and the informed consent procedure.

Cryosectioning of human LABC biopsies was performed on the Leica CM1850 Cryostat (Leica, Wetzlar, Germany) at –30°C. Sections for immunohistochemistry were cut at 10 microns and mounted on Superfrost Plus Micro Slides (VWR, West Chester, PA), and sections for metabolite bioluminescence were cut at 20 microns and mounted on 22×40 micro cover glass (VWR) coverslips.

Bioluminescence techniques were carried out as previously described [10], [36], [37], [87]. Briefly, 20 micron-thick frozen sections were exposed to a luciferase-bound LDH enzyme mixture and quickly placed in the dark under a light microscope camera. Luciferase signal was acquired for 10 seconds. Known lactate standards were run in parallel. All 16-bit images were imported to Image J for pixel grayscale intensity analysis; a standard curve was generated from the pixel values from the lactate standards. Lactate values were extrapolated from the standard curve. Three to four sections were measured per sample.

### Protein extraction and Western Blots

Protein extraction was carried out on ice using complete RadioImmuno Precipitation Assay (RIPA) lysis buffer + 0.1% protease inhibitor cocktail (BioRad, Hercules, CA). Concentrations of total protein were measured with DC™ Protein Assay from BioRad (Hercules, CA). For MCT1 Western blots, 15–50 ug per sample were loaded into wells. Proteins were separated by SDS-PAGE in a 12% gel (Bio-Rad, Hercules, CA), then transferred to a polyvinylidene fluoride (PDVF) membrane (Bio-Rad). Membranes were blocked for one hour in 5% non-fat, dry milk reconstituted in TBST. MCT1 rabbit anti-human IgG primary antibody (Millipore, Billerica, MA) and MCT4 rabbit (recognizes human, mouse and rat) IgG primary antibody (Santa Cruz Biotechnology, Dallas, TX, USA) were used, diluted 1∶1000 in TBST and incubated in 4°C overnight. The secondary goat anti-rabbit IgG horseradish peroxidase-linked antibody (Jackson ImmunoResearch Labs, West Grove, PA) was diluted 1∶2000 in TBST and incubated in room temperature for one hour. SuperSignal® West Pico Luminol/Enhancer Solution (Pierce, Rockford, IL) was used to detect bands before exposure to Kodak film (Rochester, NY). Pan-actin controls were run in accordance.

### Measurement of ^13^C lactate uptake using NMR: *in vitro studies*

Tissue culture dishes (15cm diameter) were plated at cell densities between 2 and 3×10^6^ cells. After allowing 24 h for cells to attach, or after achieving 80% confluency, cells were washed twice with 1x DPBS and then treated with either high-glucose DMEM (untreated control), no glucose (and no pyruvate) DMEM + ^12^C (unlabeled) lactate (unlabeled control) or 3-^13^C-lactate (Isotec, Sigma, St. Louis, MO). Cells were exposed to 10–40 mM 3-^13^C-lactate in normoxic or hypoxic conditions for 4, 12 or 24 h. At the end of the incubation period, 1 mL of media was collected and immediately frozen at –80°C. These media samples were used to acquire ^1^H, ^13^C and HMQC spectra of exported metabolites. Cells were washed twice with DPBS. For metabolite extraction, 1 mL of 0.9 M perchloric acid, diluted 1∶10 in diH_2_O, was added to each dish, and cells were scraped. The samples were centrifuged at 12,000 RPM for 10 minutes to pellet cell debris and precipitate. Supernatant was transferred to a fresh tube, and this was used for NMR on cell lysates.

For NMR readings, 600 µL total volume was used. For cell lysates, 450 µL of the perchloric acid cell lysate, 100 µL DPBS and 50 µL D_2_O were added to the NMR tube. For media samples, 550 µL media and 50 uL D_2_O were mixed. Tubes were capped and placed in the 500 MHz Varian Inova NMR spectrometer (Palo Alto, CA). The samples were measured with the High Resolution NMR Spectroscopy shared resource of the Duke Cancer Institute. VnmrJ Software (Varian) was used to acquire and analyze the spectra. All samples were tuned and shimmed before data acquisition. ^13^C-NMR spectra were acquired at 125.7 MHz with a Varian 500 MHz spectrometer equipped with a 5 mm broad-brand probe. Specifications were: a 45° flip angle, 0.8 s interpulse delay, and a 1.334 s acquisition time. All proton spectra were measured at 500 MHz.

### Annexin V/7-AAD staining

Cells were seeded in 6-well plates and allowed to reach 70% confluence before 24 h lactate treatment in high glucose DMEM+10% FBS + 1% antibiotic/antimycotic or glucose-free and sodium pyruvate-free DMEM+10% FBS + 1% antibiotic/antimycotic. Lactate concentrations used were 0–40 mM. For MCF7 and R3230Ac cells, treatment groups also included high (5 mM) or low (0.1 mM) CHC concentrations in the context of available glucose or glucose-deprivation and with or without 40 mM lactate. After treatment, media and cells were collected and centrifuged. Cells were resuspended in PBS and centrifuged. Live (unfixed) cells were then resuspended in 100 µL of 1X Annexin Binding Buffer (BD Pharmigen, San Diego, CA) in a round-bottom polystyrene tube (BD Falcon™, Bedford, MA). 5 µL of PE-labeled Annexin V (BD Pharmigen, San Diego, CA) and 5 µL 7-AAD (BD Pharmigen, San Diego, CA) were added to each experimental tube. Samples were incubated in the dark for 15 minutes and tapped gently to mix. 300 µL of Annexin Binding Buffer was added to each tube. Tubes were covered with aluminum foil and transported to Duke Cancer Institute Flow Cytometry Shared Facility. All sample data for cell cycle were analyzed with BD Calibur Flow Cytometer (GMI, Ramsey, MN). 10,000 events were acquired and the percentage of total cells with positive staining was reported. Error bars represent ± SEM. Results were analyzed with One-Way ANOVA and post-hoc tests.

### Animals

Female Fischer 344 rats (n = 9 for ^14^C-glucose experiments, n = 3 for ^14^C-lactate experiments) were implanted subcutaneously in dorsal mammary fat pad with 1–2 mm pieces of R3230Ac mammary carcinoma tissue extracted from a tumor from a donor animal. Experiments were conducted when tumors reached 1–2 cm in diameter. Prior to the start of the experiment rats were fasted for four hours [88]. Rats were anesthetized with isofluorane gas. Once anesthetized, the rat was placed on a heating pad (K-module, Baxter Healthcare, Valencia, CA) to maintain body temperature of 37°C. The heating pad and rat were contained within a light-tight box so that the light detected by the scintillation probes came only from interaction with the ^14^C-electrons. The femoral artery and vein were cannulated for monitoring blood pressure with a Digital Manometer (Fiber optic Sensor Technologies Inc., Ann Arbor MI) and infusion of ^14^C-glucose, ^14^C-lactate or ^13^C-lactate (*in vivo* NMR experiments).

This study was approved by Duke University’s Institutional Animal Care and Use Committee (IACUC), and the experiments were carried out in strict adherence to their guidelines and recommendations. Duke University maintains an animal program that is registered with the USDA (Permit # 83), assured through the NIH/PHS (Permit # A3195-01), and accredited with AAALAC, International (Permit # 363). All surgery was performed under isofluorane anesthesia, and all efforts were made to minimize suffering.

### ^14^C scintillation Probe Calibration

The purpose of these studies was to determine the kinetics of ^14^C-labeled lactate and ^14^C-labeled glucose uptake and excretion from tumor, compared with normal tissue. The detection device and scintillation probes were provided by Sicel Technologies Inc., Durham, NC. The apparatus consisted of fiber-optic scintillation probes connected to photomultiplier tubes (PMTs) and a computer. The electrons emitted by ^14^C were detected by the fiber-optic probes, and this interaction of the electrons with the probe creates photons that are detected by the PMTs. In principle, the apparatus works like a scintillation counter. For each experiment two probes were used (tumor and SQ of the flank). Tumor dimensions were measured and the length of probe to be inserted into the tumor was determined (6–10 mm), based on tumor size. The length of probe inserted into the subcutaneous position was always 20 mm. Each individual probe was calibrated using a ^14^C-lactate solution of known concentration (µCi/mL) with the probe being immersed in the solution at the same depth as the *in vivo* tumor measurement. This calibration was done to normalize for variability between probes and the insertion length of probe used in different tumors. Prior to the start of the probe calibration, the PMTs were turned on to check for light leakage into the device. If there was no light leakage measurements were recorded every two minutes for approximately three hours. After the calibration was finished, probes were cleaned with alcohol.

### Pharmacokinetics of ^14^C radioisotope *in vivo*

For each experiment, one probe was placed in the tumor and a second in SQ tissue. After the cover of the light-tight box was positioned a 45–60 minute probe stabilization period was allowed to elapse prior to injection of the ^14^C-labeled substrate. During this time and for the rest of the experiment the rat was continually monitored. Blood pressure and heart rate were monitored using the Digital Manometer (Fiber optic Sensor Technologies Inc., Ann Arbor MI). Body temperature was measured using a rectal probe. Breathing rate and depth of anesthesia were monitored by observation via an infrared camera inside the box connected to a small television.

After stabilization, 100 µCi of ^14^C-glucose (1 mCi/ml) or 50 µCi of ^14^C-lactate (1 mCi/ml) was infused at a rate of 0.1 mL/minute and signals were collected for approximately three hours. Time zero was defined as the start of the infusion. Blood samples (300 µL –500 µL) were taken at 0, 4, 7, 10, 15, 25, 30, 60, 100, and 160 minutes from the artery cannula using heparinized syringes. The samples were centrifuged (40,000 RPM, 15 min) and the plasma removed and weighed. 7 mL of Ultima Gold scintillation cocktail (Perkin Elmer Life and Analytical Sciences Inc., Boston MA) was added to each vial. Samples were measured in a Packard Tri-Carb 1500 Liquid Scintillation Counter (Perkin Elmer Life and Analytical Sciences Inc., Boston MA). Counts per minute (CPM) were converted to Disintegrations per Minute (DPM) by the scintillation counter by using set of ^14^C-standards at known concentrations. The scintillation counter uses the standards to create a quench curve allowing for the conversion of CPMs to DPMs. Concentration (μCi/g) for each sample was calculated by dividing the number of µCis in the plasma by plasma weight. The blood activity data were then normalized to dose/body weight.

### Compartmental model

^14^C-glucose and ^14^C-lactate uptake data were analyzed using compartmental modeling. A 3-compartmental model was used for ^14^C-glucose and ^14^C-lactate data. The following parameters were defined in the model: C_p_  =  glucose or lactate concentration in the blood compartment, C_i_  =  glucose or lactate concentration in the tumor compartment, and C_s_  =  glucose or lactate concentration in the subcutaneous compartment. Rates: k_0_  =  clearance by other tissues, k_1_  =  transfer rate of glucose or lactate from blood into tumor, k_2_  =  transfer rate out of tumor, k_3_  =  transfer rate into the subcutaneous tissue, and k_4_  =  transfer rate out of the subcutaneous tissue. The details of the model are described in the supplemental material (Fig S1).

### ^13^C lactate NMR from extracted R3230Ac tumors

For *in vivo* NMR experiments, rats were handled in a similar manner. The femoral artery and vein were cannulated for monitoring blood pressure with Digital Manometer (Fiber optic Sensor Technologies Inc., Ann Arbor MI) and infusion of ^13^C-lactate, respectively. 1 mL of 100 mM ^13^C-lactate was infused intravenously over 10 minutes. Time zero was considered to be the start of the infusion. At baseline, 15, 30, 45, 60 and 90 minutes rats were sacrificed and the tumor, liver and brain removed within 60 s and snap-frozen in liquid nitrogen. The frozen tissues were pulverized with a mortar and pestle under liquid nitrogen. 2 mL total of 0.9 M perchloric acid was added to the pulverized tissue. Once thawed at 4°C, the extract was homogenized and neutralized with KOH. Salt was removed by centrifugation and the supernatant was frozen and lyophilized. Each experiment was repeated three times.

The lyophilized samples were dissolved in 630 µL phosphate buffer and 70 µL (10%) deuterium. 15 µL of dioxane was added to each sample as an internal standard. ^13^C-NMR spectra were acquired at 125.7 MHz with a Varian 500 MHz spectrometer equipped with a 5 mm broad-brand probe. A 45° flip angle, 0.8 s interpulse delay, and a 1.334 s acquisition time were used for all experiments. Standards of glucose, G6P, glutamate, lactate, and alanine at concentrations of 1 mM, 5 mM, and 10 mM with dioxane as an internal standard were also run. The standards were used to create standard curves, which were used to convert peak heights of the different metabolites in each spectrum to concentrations. Additionally the data were analyzed, comparing ratios of different metabolites within a spectrum. The ratios were obtained from peak heights of metabolites.

### Autoradiography

^14^C-lactate autoradiography was used to study the spatial distribution of lactate uptake. Athymic nude mice transplanted with R3230Ac tumors were infused with twoµCi of ^14^C-lactate. The hypoxia marker drug, pimonidazole (60 mg/kg i.p.), was injected. Thirty minutes later, Hoechst-33342 was administered intravenously as a perfusion marker dye (10 mg/ml, 0.05 ml). Two minutes later, tissues were harvested and snap-frozen. A portion of the frozen tumor was cryosectioned at 14 µm thickness for autoradiography, Hoechst staining and pimonidazole staining.

Electronic autoradiography was performed with a storage phosphor system (Packard Bioscience, Downers Grove, IL). Sections were loaded onto a storage phosphor screen and exposed for 3 weeks. A slide with ^14^C-standards was also placed on the screen for quantification. The screen was then processed and read in the phosphor system to visualize the distribution of ^14^C-lactate. Intensity level obtained from the reader was converted to activity, based on the standard slides.

### Immunohistochemistry staining

To co-localize the lactate uptake and oxygenation status in tumor, immunostaining of hypoxia with pimonidazole was performed on tumor sections. Tumor cryosections were fixed with paraformaldehyde (room temperature, 30 minutes), blocked, and then stained with mouse anti-pimonidazole antibody labeled with Alexa Fluor® 555 (excitation wavelength  =  580 nm). Slides were imaged using a microscope equipped with automatic scanning stage for whole slide scan. Hoechst-33342 (excitation wavelength  =  460 nm) images were also obtained by scanning slide with blue filter.

Autoradiography, hypoxia, and perfusion images were registered and overlaid to show the relationship between lactate uptake, hypoxia, and perfusion.

### Measurement of oxygen consumption in confluent cells using a Seahorse XF24 extracellular flux analyzer

R3230Ac cells, cultured in high glucose DMEM with 10% serum and 1% antibiotics were washed, trypsinized, counted, and seeded in Seahorse XF24 cell culture plates at 60,000 cells per well in growth medium. According to the manufacturer’s instructions, cells were allowed to adhere for 2 hours before growth medium was added to a total volume of 250 µL. Cells were allowed to grow overnight at 37°C, 5% CO_2_. On the day of the experiment, cells were washed and mounted with warm XF assay medium, and incubated for 60 min at 37°C without CO_2_ before starting the experiment. During the experiment, Na-Lac was added to each well at varying concentrations (diluted in water) by the automatic dispenser to equal a total volume of 75 µL. Three independent experiments were carried out in triplicate. Results are represented as percent of baseline respiration rate 4 minutes after addition of lactate.

### Statistics

Student’s T-test was used to evaluate mean lactate concentration differences between duplicate LABC biopsies from the same patient. Student’s T-test was also used to evaluate differences in: 1. Oxygen consumption in lactate-treated cell in Figure 2, 2. Cell number between MCF7 and MDA-MB-231 cells after five days of lactate treatment in Figure 4, and 3. Cell number between glucose-free, lactate supplemented media and high-glucose, lactate-free media in Figure S5. A linear regression analysis was applied to cellular lactate consumption data in Figure 4. One-way analysis of variance (ANOVA) and Bonferroni/Dunn post-hoc tests were used to evaluate the main effects of lactate treatment on Annexin V/7-AAD staining for the cell lines tested. Cell viability statistics were analyzed with StatView software. All error bars on plots express ± SEM.

## Supporting Information

Figure S1 **24 h exposure to high lactate concentrations do not decrease cell viability or increase cell death responses when glucose is available** ***in vitro*** **.** Cell viability as measured by Annexin V −/7-AAD – labeling (n = 3) in normal human mammary epithelial cells (HMEC) (**A**), MCF7 (**B**) MDA-MB-231 (**C**) and R3230Ac cells (**D**) show no significant changes 24 h after addition of exogenous sodium lactate (0−40 mM) in the context of available glucose. No significant changes in cell death responses (Annexin V +/7-AAD –, Annexin V −/7-AAD +, or Annexin V+/7-AAD +) (n = 3) were observed in HMEC (**E**), MCF7 (**F**) MDA-MB-231 (**G**) and R3230Ac cells (**H**) after addition of exogenous sodium lactate (0-40 mM) in the context of available glucose.
(TIF)

Figure S2 **24 h exposure to high lactate concentrations (-glucose) significantly decrease breast cancer cell viability and increase cell death responses in human breast cancer cells but not normal breast or R3230Ac cells** ***in vitro*** **.** Cell viability as measured by Annexin V −/7-AAD – labeling (n = 3) in normal human mammary epithelial cells (HMEC) (**A**) and R3230Ac cells (**D**) show no significant change after exposure to exogenous lactate for 24 h. MCF7 (**B**) and MDA-MB-231 cells (**C**) show a significant decrease in unstained cells after addition of 40 mM exogenous sodium lactate in the context of available glucose (One-Way ANOVA, Bonferroni/Dunn post-hoc test, # p≤0.0006 compared to untreated control and all other treatment groups). No significant changes in any cell death response were seen in HMEC (**E**) or R3230Ac cells (**H**) after lactate treatment in the context of glucose-deprivation. The percentage of cells with Annexin V+/7-AAD + labeling was significantly increased in MCF7 (**F**) and MDA-MB-231 (**G**) cells after addition of 40 mM sodium lactate with glucose deprivation (n = 3, One-Way ANOVA, Bonferroni/Dunn post-hoc test, *p < 0.0001 compared untreated control and to all other treated groups).
(TIF)

Figure S3 **MCT1 expression in breast cell lines.** Total protein expression of MCT1 in MCF7, MDA-MB-231 and HMEC cells show MCT1 expression in MCF7 and HMEC but not MDA-MB-231 cells (**A**). Total protein expression of MCT4 in HMEC, MDA-MB-231, MCF7 and R3230Ac cells show abundant MCT4 expression in MDA-MB-231 cells, low MCT4 expression in HMEC and no detectable MCT4 expression in MCF7 or R3230Ac cells (**B**).
(TIF)

Figure S4 **Compartmental model for ^14^C-labeled glucose and lactate to analyze** ***in vivo*** **kinetic data.** C_p_  =  glucose or lactate in the blood/plasma compartment, C_i_  =  glucose or lactate in the tumor compartment, C_s_  =  glucose or lactate in the SQ compartment. k_0_  =  clearance by other tissues, k_1_  =  transfer rate into the tumor, k_2_  =  transfer rate out of the tumor, k_3_  =  transfer rate into the subcutaneous tissue, k_4_  =  transfer rate out of the subcutaneous tissue.
(TIF)

Figure S5 **MCF7 and MDA-MB-231 cell growth in glucose-free, lactate-supplemented media vs. lactate-free, high-glucose media.** MCF7 and MDA-MB-231 cells plated at equal densities and allowed to grow for 5 days in either glucose-free, 20 mM lactate-supplemented media (n = 6) or high-glucose media (n = 3). Five days after the media change, cells were harvested and counted. MCF7 cell counts showed no difference between media, but MDA-MB-231 cell counts were significantly higher in the high-glucose (no lactate) media than in the glucose-free, 20 mM lactate media (p = 0.005, Student’s T-test).
(TIF)

Figure S6 **Normal human cells take up lactate and export catabolites.** HMQC plots of HMEC cell lysate (**A**) and media (**B**) after 4.5 h incubation with 10 mM ^13^C-lactate, showing evidence of labeled lactate (dark green “**L**”) and glutamate (blue “**G**”) peaks. 1H spectra of HUVEC cell lysate (bottom) and media (top) after 24 h treatment with 5 mM ^13^C-lactate (**C**). The lysate spectrum shows peaks corresponding to lactate, indicating uptake; the media spectrum shows evidence of labeled lactate, alanine and glutamate.
(TIF)

Figure S7 **Control NMR spectra for R3230Ac cell media alone or treated with no lactate + high or low CHC.** ^13^C spectrum of glucose-free, pyruvate-free, +glutamine (+10% FBS) DMEM used for all *in vitro* NMR experiments show low background levels of ubiquitous metabolites (**A**). ^1^H (**B&C**) or ^13^C (**D&E**) spectra of R3230Ac cell lysates (**B&C**) or media (**D&E**) incubated for 4 h with no labeled lactate, no glucose + 0.1 mM CHC (**B&D**) or 5 mM CHC (**C&E**), showing no labeled lactate.
(TIF)

Figure S8 **^1^H NMR spectra of R3230Ac cell media treated with ^13^C-lactate + CHC show evidence of exportation of some endogenous lactate.** ^1^H spectra of R3230Ac cell media incubated with 20 mM ^13^C-lactate and 0.1 mM (**A&C**) or 5 mM (**B&D**) of CHC for 4 h (**A&B**) or 24 h (**C&D**) show an abundance of ^13^C-lactate and incomplete inhibition of endogenous lactate (green “**L_en_**”) exportation.
(TIF)
