# Supplementary figures and images for: Catabolism of Exogenous Lactate Reveals It as a Legitimate Metabolic Substrate in Breast Cancer
Source: PLoS One. 2013 Sep 12;8(9):e75154. doi: 10.1371/journal.pone.0075154 (PMC3771963; doi:10.1371/journal.pone.0075154)

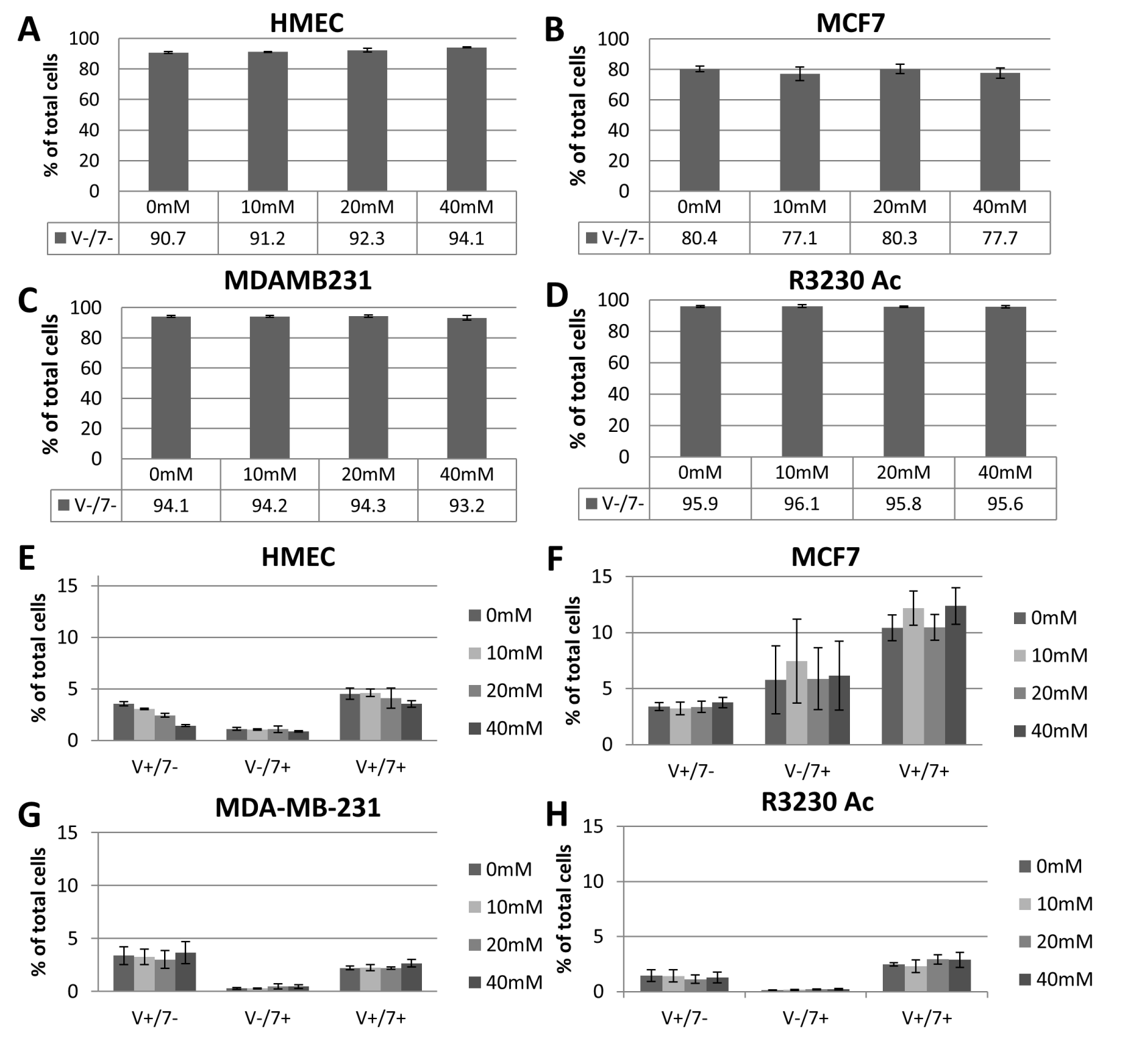

Supplement: Figure S1 — 24 h exposure to high lactate concentrations do not decrease cell viability or increase cell death responses when glucose is available in vitro . Cell viability as measured by Annexin V −/7-AAD – labeling (n = 3) in normal human mammary epithelial cells (HMEC) (A), MCF7 (B) MDA-MB-231 (C) and R3230Ac cells (D) show no significant changes 24 h after addition of exogenous sodium lactate (0−40 mM) in the context of available glucose. No significant changes in cell death responses (Annexin V +/7-AAD –, Annexin V −/7-AAD +, or Annexin V+/7-AAD +) (n = 3) were observed in HMEC (E), MCF7 (F) MDA-MB-231 (G) and R3230Ac cells (H) after addition of exogenous sodium lactate (0-40 mM) in the context of available glucose. (TIF) [file pone.0075154.s001.tif]

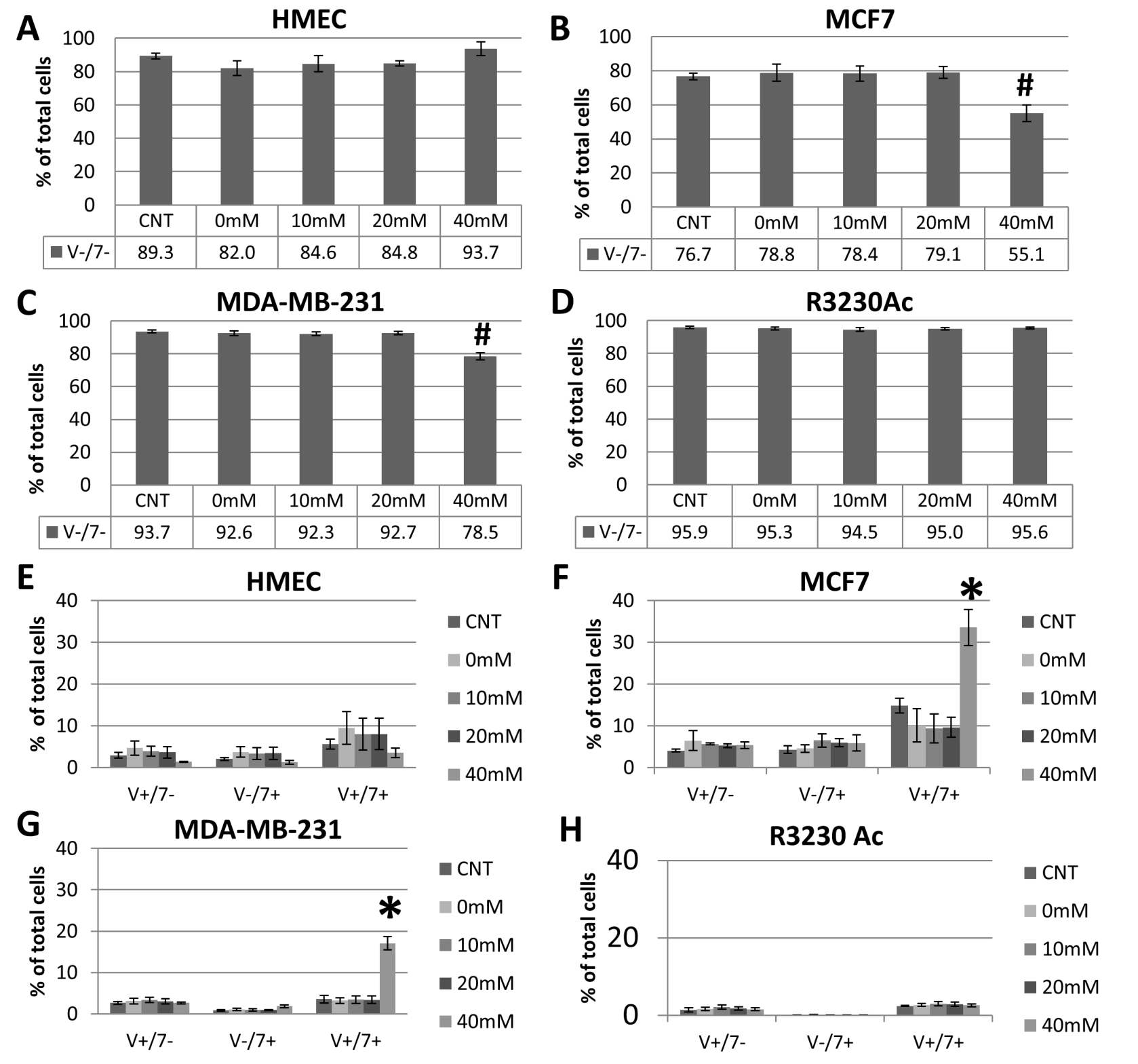

Supplement: Figure S2 — 24 h exposure to high lactate concentrations (-glucose) significantly decrease breast cancer cell viability and increase cell death responses in human breast cancer cells but not normal breast or R3230Ac cells in vitro . Cell viability as measured by Annexin V −/7-AAD – labeling (n = 3) in normal human mammary epithelial cells (HMEC) (A) and R3230Ac cells (D) show no significant change after exposure to exogenous lactate for 24 h. MCF7 (B) and MDA-MB-231 cells (C) show a significant decrease in unstained cells after addition of 40 mM exogenous sodium lactate in the context of available glucose (One-Way ANOVA, Bonferroni/Dunn post-hoc test, # p≤0.0006 compared to untreated control and all other treatment groups). No significant changes in any cell death response were seen in HMEC (E) or R3230Ac cells (H) after lactate treatment in the context of glucose-deprivation. The percentage of cells with Annexin V+/7-AAD + labeling was significantly increased in MCF7 (F) and MDA-MB-231 (G) cells after addition of 40 mM sodium lactate with glucose deprivation (n = 3, One-Way ANOVA, Bonferroni/Dunn post-hoc test, *p < 0.0001 compared untreated control and to all other treated groups). (TIF) [file pone.0075154.s002.tif]

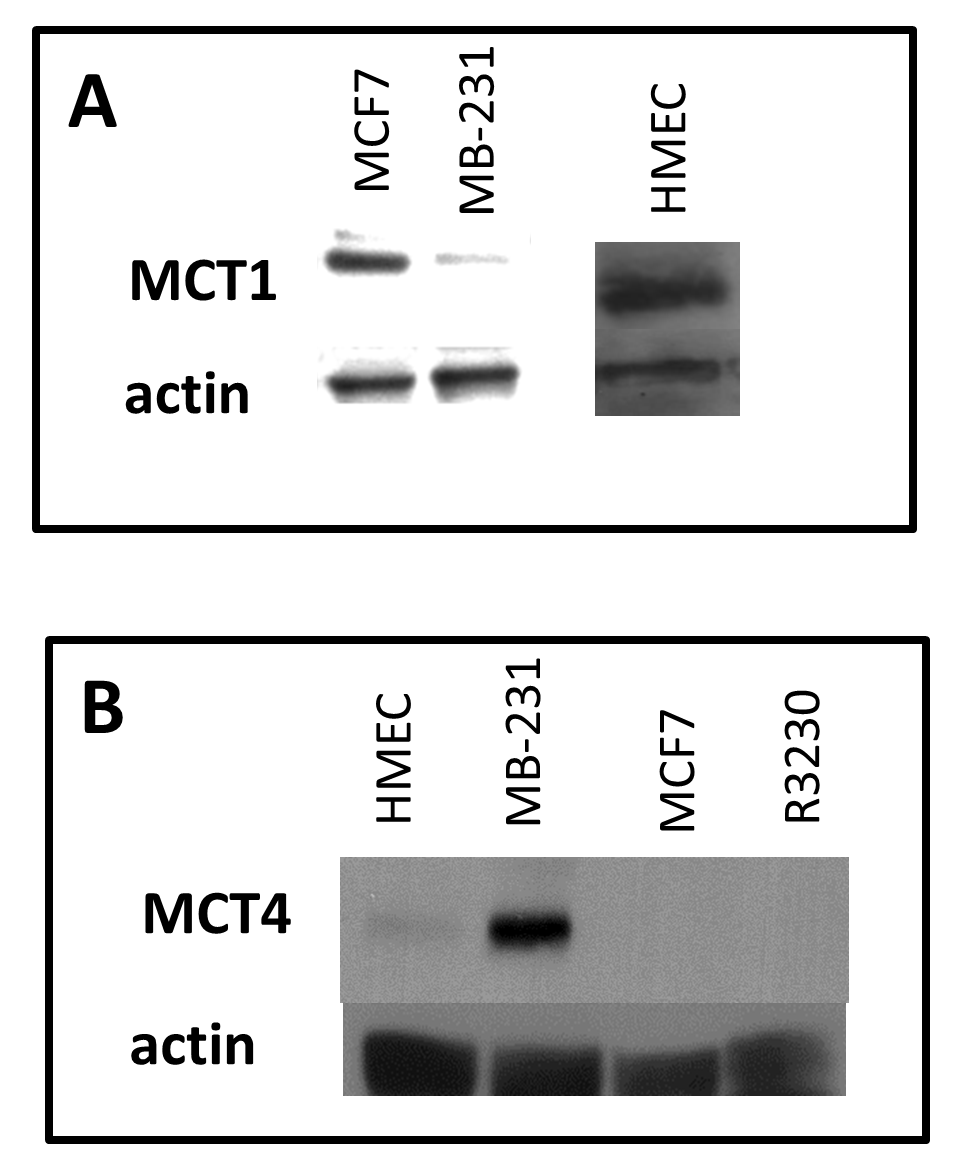

Supplement: Figure S3 — MCT1 expression in breast cell lines. Total protein expression of MCT1 in MCF7, MDA-MB-231 and HMEC cells show MCT1 expression in MCF7 and HMEC but not MDA-MB-231 cells (A). Total protein expression of MCT4 in HMEC, MDA-MB-231, MCF7 and R3230Ac cells show abundant MCT4 expression in MDA-MB-231 cells, low MCT4 expression in HMEC and no detectable MCT4 expression in MCF7 or R3230Ac cells (B). (TIF) [file pone.0075154.s003.tif]

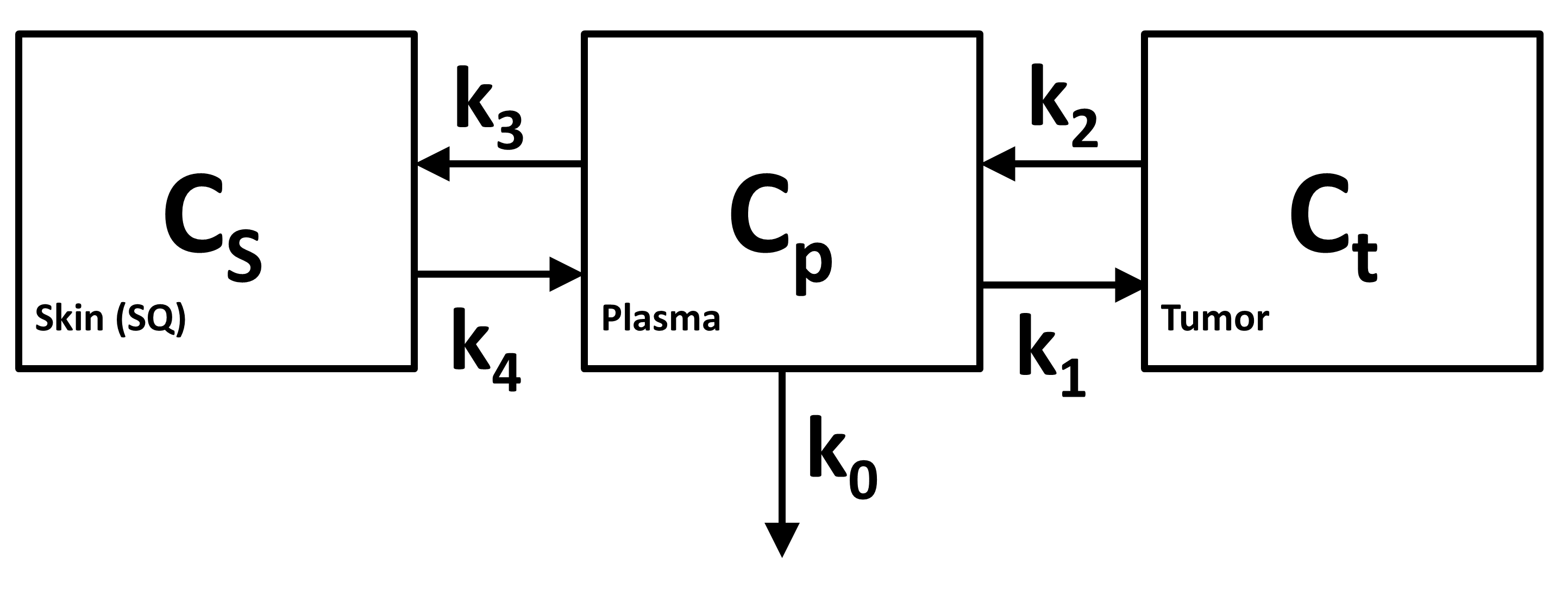

Supplement: Figure S4 — Compartmental model for 14C-labeled glucose and lactate to analyze in vivo kinetic data. Cp = glucose or lactate in the blood/plasma compartment, Ci = glucose or lactate in the tumor compartment, Cs = glucose or lactate in the SQ compartment. k0 = clearance by other tissues, k1 = transfer rate into the tumor, k2 = transfer rate out of the tumor, k3 = transfer rate into the subcutaneous tissue, k4 = transfer rate out of the subcutaneous tissue. (TIF) [file pone.0075154.s004.tif]

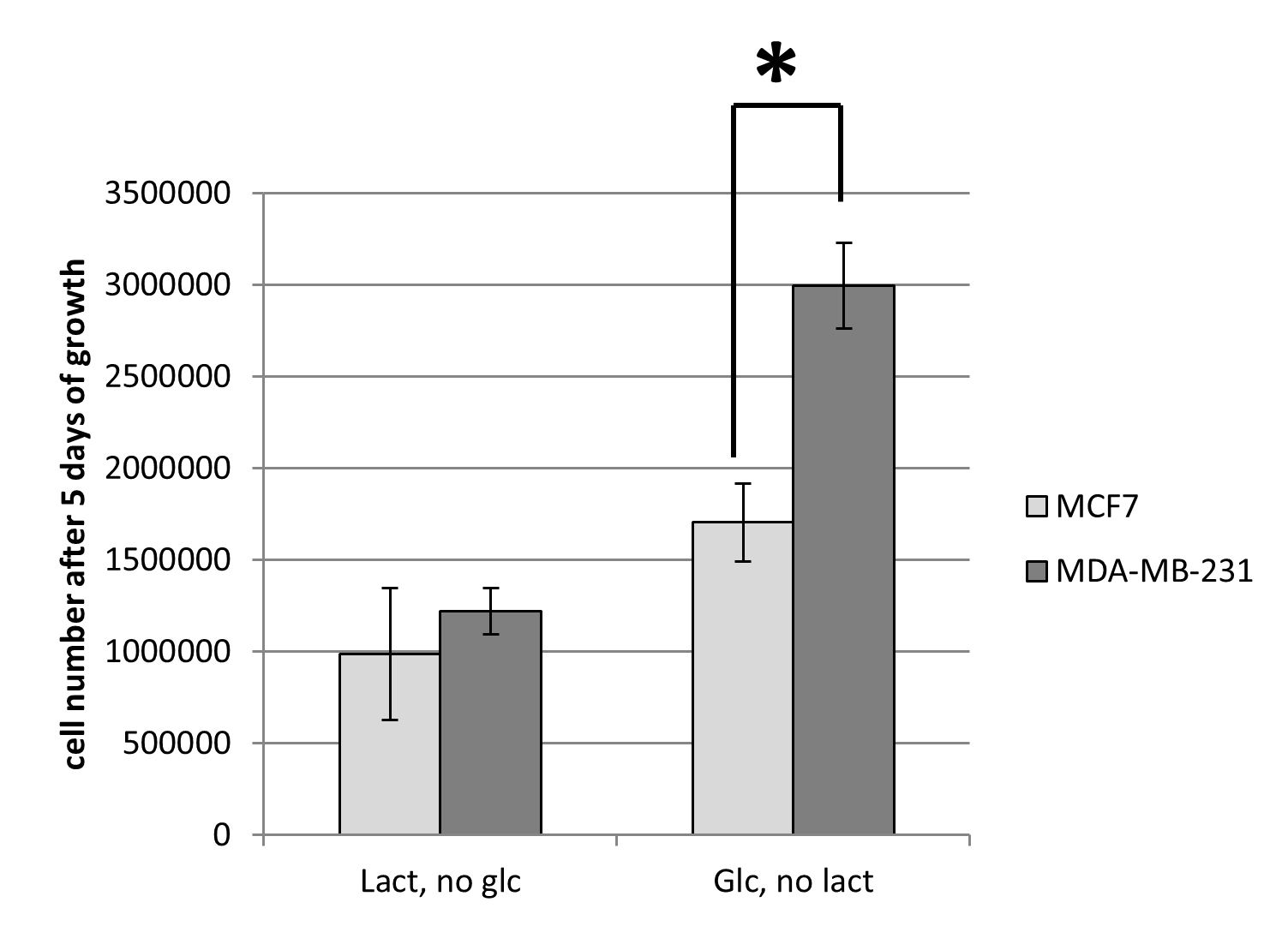

Supplement: Figure S5 — MCF7 and MDA-MB-231 cell growth in glucose-free, lactate-supplemented media vs. lactate-free, high-glucose media. MCF7 and MDA-MB-231 cells plated at equal densities and allowed to grow for 5 days in either glucose-free, 20 mM lactate-supplemented media (n = 6) or high-glucose media (n = 3). Five days after the media change, cells were harvested and counted. MCF7 cell counts showed no difference between media, but MDA-MB-231 cell counts were significantly higher in the high-glucose (no lactate) media than in the glucose-free, 20 mM lactate media (p = 0.005, Student’s T-test). (TIF) [file pone.0075154.s005.tif]

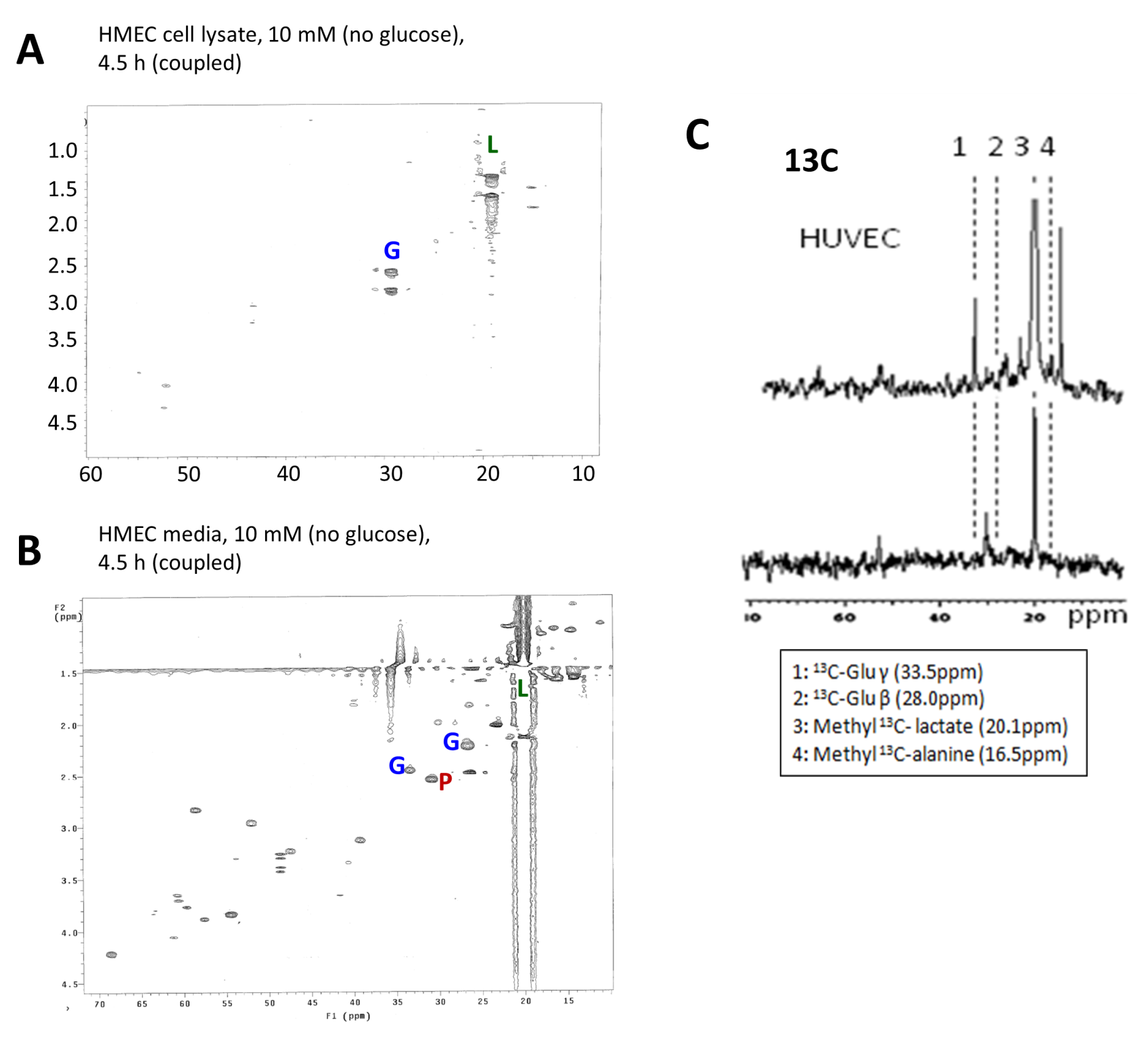

Supplement: Figure S6 — Normal human cells take up lactate and export catabolites. HMQC plots of HMEC cell lysate (A) and media (B) after 4.5 h incubation with 10 mM 13C-lactate, showing evidence of labeled lactate (dark green “L”) and glutamate (blue “G”) peaks. 1H spectra of HUVEC cell lysate (bottom) and media (top) after 24 h treatment with 5 mM 13C-lactate (C). The lysate spectrum shows peaks corresponding to lactate, indicating uptake; the media spectrum shows evidence of labeled lactate, alanine and glutamate. (TIF) [file pone.0075154.s006.tif]

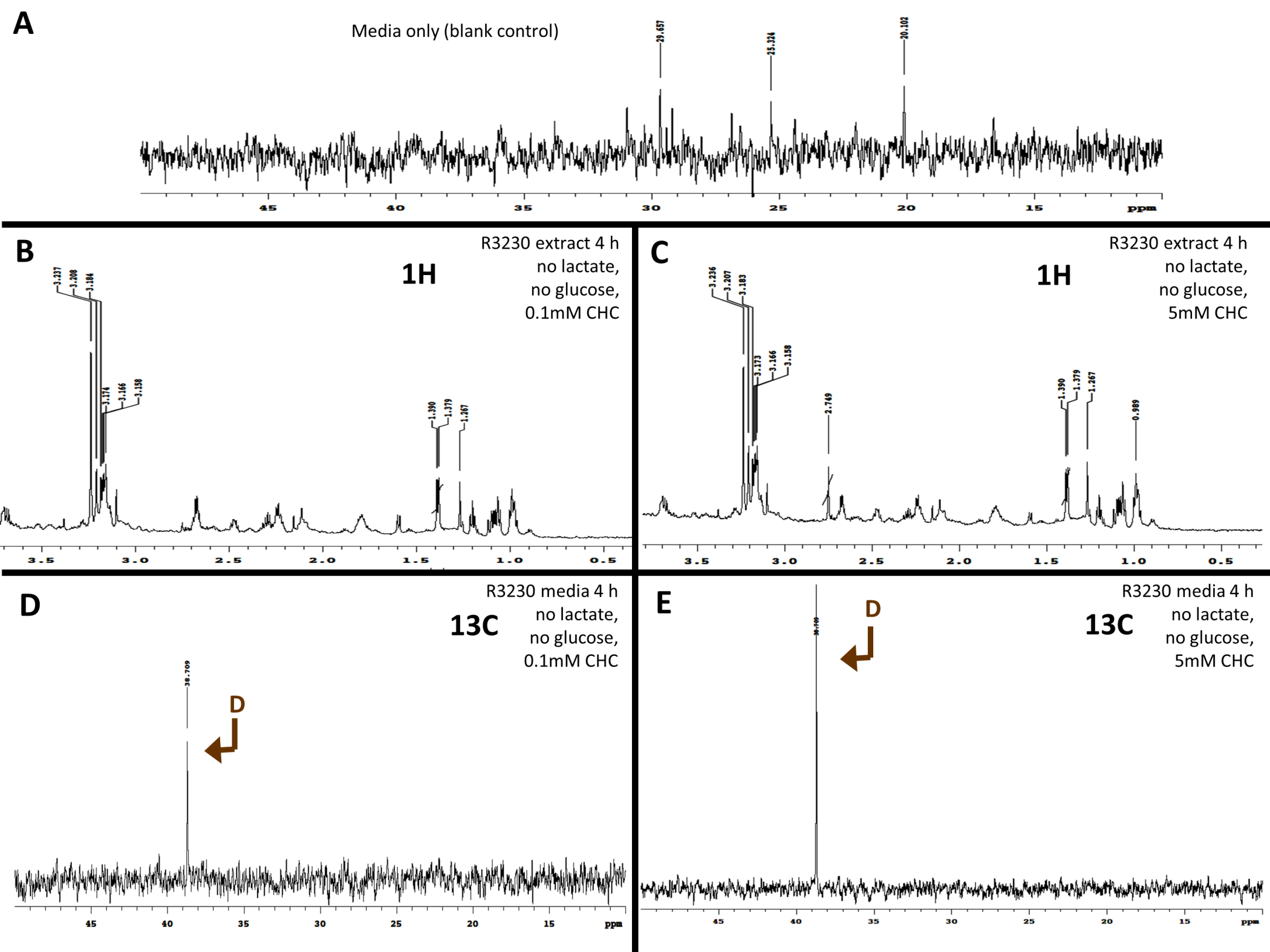

Supplement: Figure S7 — Control NMR spectra for R3230Ac cell media alone or treated with no lactate + high or low CHC. 13C spectrum of glucose-free, pyruvate-free, +glutamine (+10% FBS) DMEM used for all in vitro NMR experiments show low background levels of ubiquitous metabolites (A). 1H (B&C) or 13C (D&E) spectra of R3230Ac cell lysates (B&C) or media (D&E) incubated for 4 h with no labeled lactate, no glucose + 0.1 mM CHC (B&D) or 5 mM CHC (C&E), showing no labeled lactate. (TIF) [file pone.0075154.s007.tif]

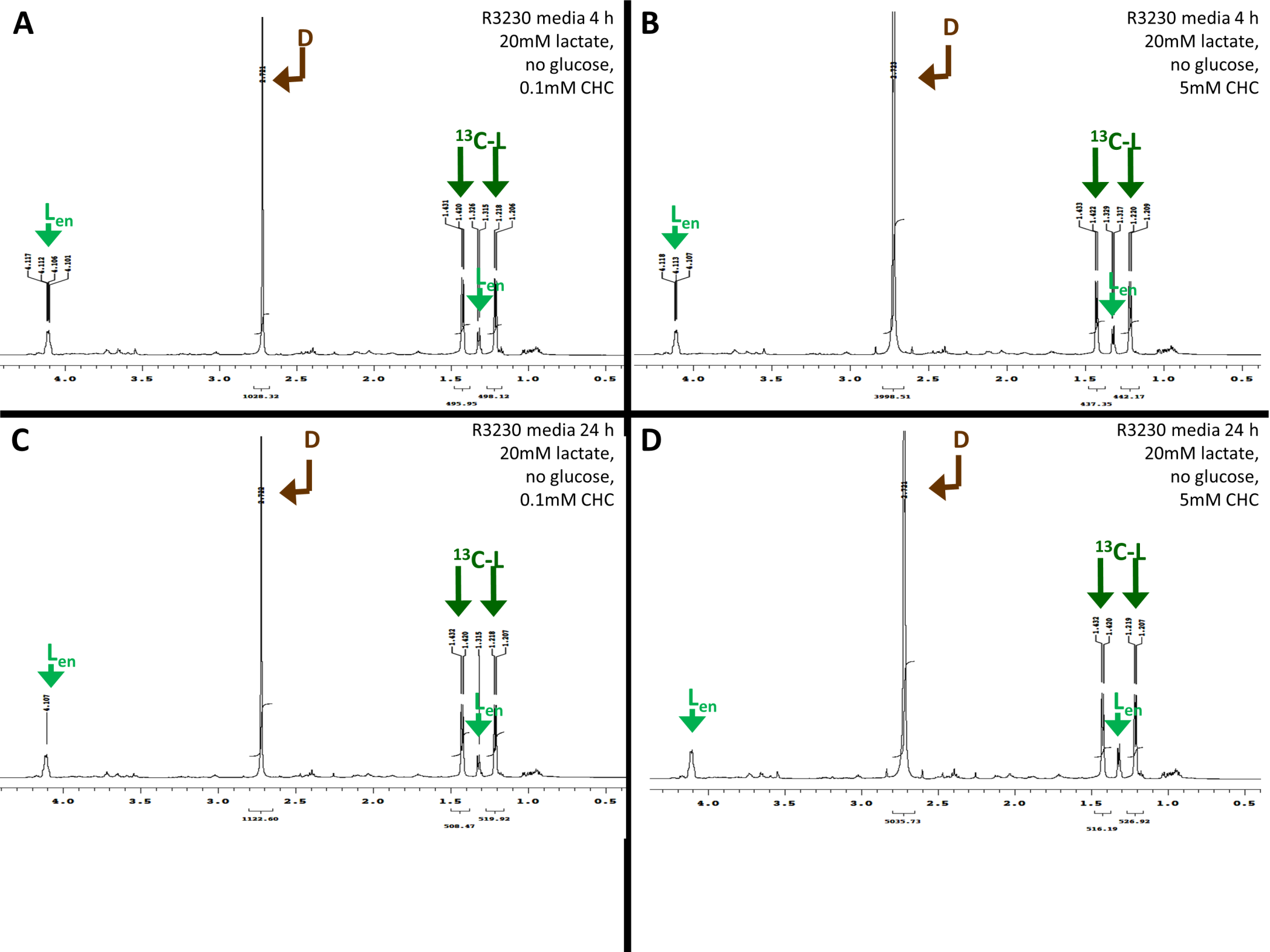

Supplement: Figure S8 — 1H NMR spectra of R3230Ac cell media treated with 13C-lactate + CHC show evidence of exportation of some endogenous lactate. 1H spectra of R3230Ac cell media incubated with 20 mM 13C-lactate and 0.1 mM (A&C) or 5 mM (B&D) of CHC for 4 h (A&B) or 24 h (C&D) show an abundance of 13C-lactate and incomplete inhibition of endogenous lactate (green “Len”) exportation. (TIF) [file pone.0075154.s008.tif]
